# Radiomics-based neural network predicts recurrence patterns in glioblastoma using dynamic susceptibility contrast-enhanced MRI

**DOI:** 10.1038/s41598-021-89218-z

**Published:** 2021-05-11

**Authors:** Ka Young Shim, Sung Won Chung, Jae Hak Jeong, Inpyeong Hwang, Chul-Kee Park, Tae Min Kim, Sung-Hye Park, Jae Kyung Won, Joo Ho Lee, Soon-Tae Lee, Roh-Eul Yoo, Koung Mi Kang, Tae Jin Yun, Ji-Hoon Kim, Chul-Ho Sohn, Kyu Sung Choi, Seung Hong Choi

**Affiliations:** 1 Seoul National University College of Medicine, Seoul, Republic of Korea; 2 Department of Radiology, Seoul National University Hospital, 101 Daehangno, Jongno-gu, Seoul, 110-744 Republic of Korea; 3 Department of Neurosurgery and Biomedical Research Institute, Seoul National University Hospital, Seoul, Korea; 4 Department of Internal Medicine and Cancer Research Institute, Seoul National University Hospital, Seoul, Korea; 5 Department of Pathology, Seoul National University Hospital, Seoul, Korea; 6 Department of Radiation Oncology and Cancer Research Institute, Seoul National University Hospital, Seoul, Korea; 7 Department of Neurology, Seoul National University Hospital, Seoul, Korea; 8 Center for Nanoparticle Research, Institute for Basic Science (IBS), Seoul, Republic of Korea

**Keywords:** Cancer imaging, Cancer, Prognostic markers, Biomedical engineering, Machine learning

## Abstract

Glioblastoma remains the most devastating brain tumor despite optimal treatment, because of the high rate of recurrence. Distant recurrence has distinct genomic alterations compared to local recurrence, which requires different treatment planning both in clinical practice and trials. To date, perfusion-weighted MRI has revealed that perfusional characteristics of tumor are associated with prognosis. However, not much research has focused on recurrence patterns in glioblastoma: namely, local and distant recurrence. Here, we propose two different neural network models to predict the recurrence patterns in glioblastoma that utilizes high-dimensional radiomic profiles based on perfusion MRI: area under the curve (AUC) (95% confidence interval), 0.969 (0.903–1.000) for local recurrence; 0.864 (0.726–0.976) for distant recurrence for each patient in the validation set. This creates an opportunity to provide personalized medicine in contrast to studies investigating only group differences. Moreover, interpretable deep learning identified that salient radiomic features for each recurrence pattern are related to perfusional intratumoral heterogeneity. We also demonstrated that the combined salient radiomic features, or “radiomic risk score”, increased risk of recurrence/progression (hazard ratio, 1.61; *p* = 0.03) in multivariate Cox regression on progression-free survival.

## Introduction

Glioblastoma (GBM) remains the most aggressive primary brain tumor, with a median survival of 12–15 months despite optimal treatment^1^. The poor prognosis is due to the high rate of recurrence/progression^2^. In regions of physically disrupted blood brain barrier (BBB) by tumor cells, the contrast agents diffuse out of the vessels, manifesting enhancing lesions on contrast-enhanced T1-weighted images (CE T1WI) in nearly all GBM. These enhancing lesions are associated with dense tumor cells, and are the target for surgical resection^3^. However, CE T1WI is insufficient to distinguish paucicellular involvement of tumor from peritumoral edema, which is well-demonstrated on T2-weighted images (T2WI). T2 hyperintense area surrounding enhancing lesions on CE T1WI, should be considered as brain parenchyma infiltrated by isolated tumor cells, and radiation field should cover the area when planning radiation treatment^4^.

GBMs have high intratumoral heterogeneity (ITH), and result in inevitable recurrence^5^. Thus, many attempts have been made to predict relapse, or progression, and the prognosis of a tumor. Radiomics that extracts quantitative features from radiographic images is the latest approach, and many studies have been conducted to relate radiomic profiles to GBM’s molecular subtypes, genetic mutations, and/or survivals^6–10^. Among them, radiomic features obtained from cerebral blood volume (CBV) maps, or hemodynamic parameter maps derived from dynamic susceptibility contrast-enhanced MRI (DSC MRI), have been reported as potential biomarkers to predict the prognosis of GBM^11^.

Recent surgical advances has increased rate of complete resection (CR) of contrast-enhancing lesions with the help of 5-aminolevulinic acid (5-ALA) guided resection, and intraoperative neurophysiologic monitoring, which leads to improved outcome^12^. However, GBM recurrence/progression is still almost inevitable, and further investigation for effective treatment strategy is highly required for recurrent GBM^13^. Distant recurrence (DR) has been known to have different tumor biology from local recurrence (LR), which represents low rate of retention of initial tumor mutations^14^, and thus requires different treatment strategy compared to LR. However, not much study has focused on the recurrence patterns in GBM. Moreover, no study has focused on the prediction of recurrence patterns after maximal surgical resection, especially at an individual level.

Here, we developed and validated the prediction model for recurrence patterns in GBM based on perfusion radiomics. We also identified salient radiomic features obtained from CBV maps to predict the recurrence patterns using interpretable deep learning, and analyzed whether the salient features are associated with prognosis. Moreover, we examined whether the genetic difference affects the recurrence of glioblastoma. This creates an opportunity to provide personalized medicine, leading to optimal management in patients with glioblastoma.

## Results

### Patient characteristics

**Figure 1:**
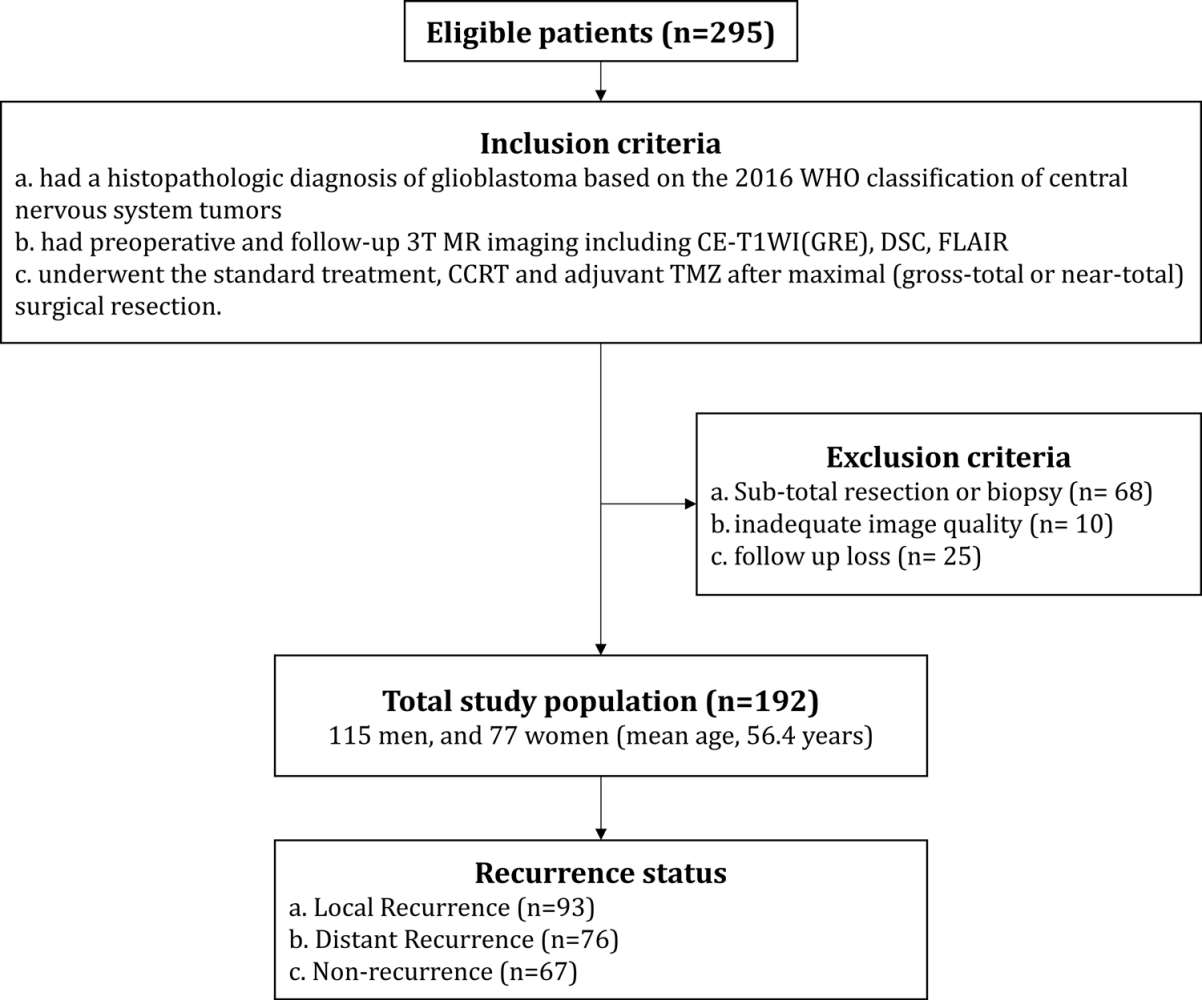
Patient inclusion/exclusion criteria.

Under the inclusion and exclusion criteria, total 192 patients were finally included in our study (Fig. 1). The clinical characteristics of the GBM patients in the disease recurrence (*n* = 125) and the non-recurrence (*n* = 67) group are summarized in Table 1. There were no differences between the two groups in regards of the age, and radiation dose. The male patients were more in the recurrence group than in non-recurrence group (86 of 125 vs. 29 of 67, respectively, *p* < 0.001). O6-Methylguanine-DNA methyltransferase (*MGMT*) promotor methylation was more frequently observed in the non-recurrence group than in recurrence group (44 of 67 vs. 50 of 125, respectively, *p* < 0.001). Isocitrate dehydrogenase (*IDH*) wildtype was more frequent in the recurrence group than the non-recurrence group (121 of 125 vs. 57 of 67, respectively, *p* = 0.01). The mean duration of LR and DR were 416 ± 298 (range 22–1666) and 342 ± 189 (range 22–1,042) days respectively, which resulted in no statistical significance (*p* = 0.051).

**Table 1:** Clinical characteristics of the study population.

Characteristics	Total (*n* = 192)	Recurrence (*n* = 125)	Non-recurrence (*n* = 67)	*p* value
**Mean age (years)**	60.2	60.8 ± 13.5	59.1 ± 12.8	0.37*
**Mean radiation dose (Gy)**	56.6	56.3 ± 7.7	57.2 ± 7.0	0.42*
**Sex**				< 0.001^†^
Male	115	86	29	
Female	77	39	38	
**Methylated MGMT promoter**				< 0.001^†^
Positive	94	50	44	
Negative	98	75	23	
***IDH1/2 mutation***				0.01^†^
Positive	14	4	10	
Negative	178	121	57	

Unless otherwise specified, data are given as the number of patients. Data are expressed as mean ± standard deviation; *MGMT*, O6-Methylguanine-DNA methyltransferase; *IDH*, Isocitrate dehydrogenase.

*Calculated with an unpaired Student’s t test.

^†^Calculated with Fisher’s exact.

### Image analysis

**Figure 2:**
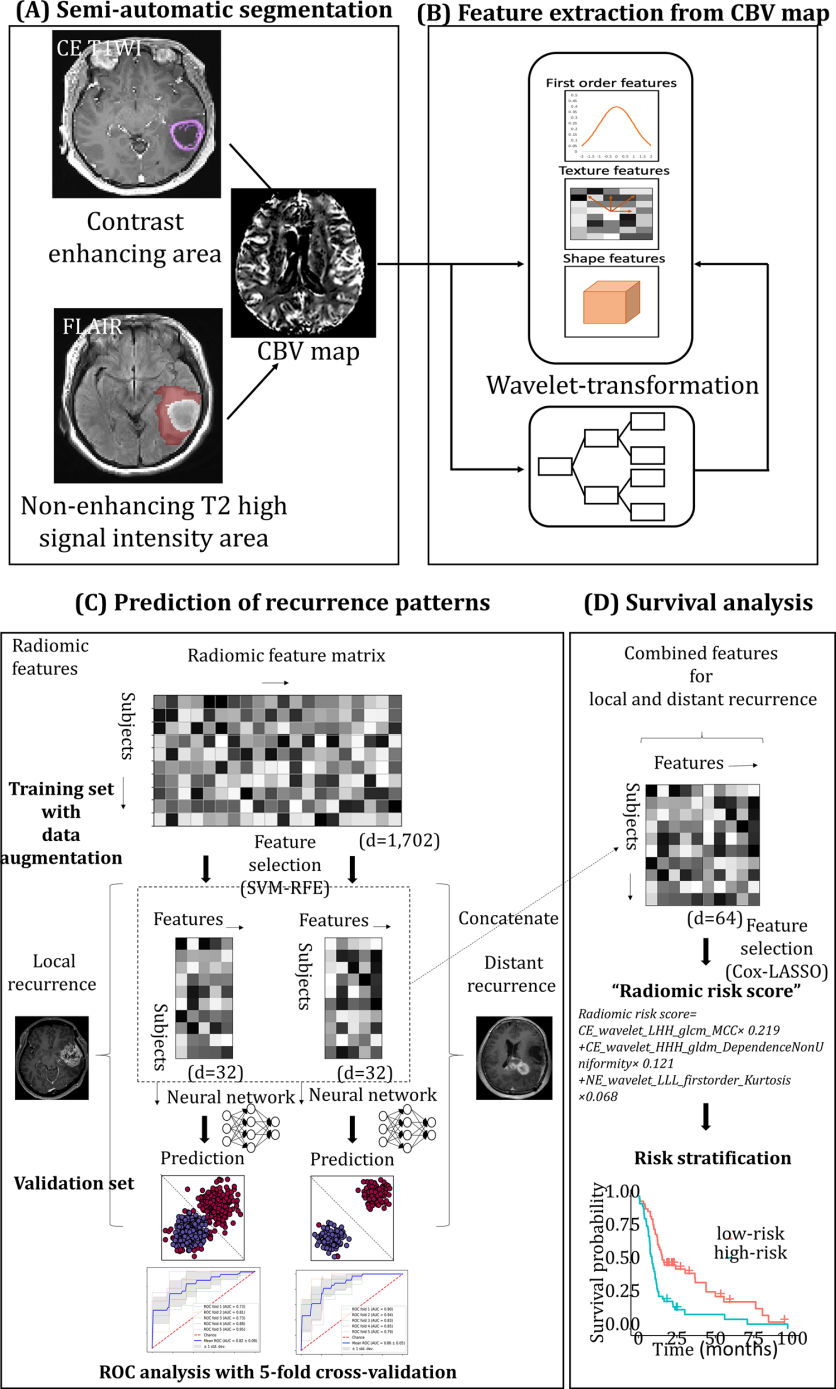
Overall workflow from tumor segmentation to prediction of recurrence patterns, and survival analysis. (**A**) Segmentation of contrast-enhanced and non-enhancing T2 hyperintense areas. (**B**) Multiple radiomic profiles including first-order, textural, shape and wavelet-transformed features were automatically calculated from contrast-enhanced and non-enhancing T2 hyperintense areas based on CBV map. Radiomic feature matrix (subjects × features) was obtained from image processing. (**C**) Two multilayer perceptron models were trained and validated to predict local and distant recurrence of glioblastoma, respectively. The prediction models were developed based on 32 features each, which were selected using SVM-RFE among 1702 features of the radiomic feature matrix. (**D**) The three selected features from the 64 features in the multilayer perceptron models using Cox-LASSO were used to develop “radiomic risk score”. The developed radiomic risk score was subjected to Cox proportional hazard model in addition to clinical variables to regress the progression free survival (PFS). *CBV* cerebral blood volume, *SVM-RFE* recursive feature elimination with support vector machine, *Cox-LASSO* Cox regression with least absolute shrinkage and selection operator.

For perfusion radiomics, cerebral blood volume (CBV) maps were generated from DSC MRI. For each patient, total 1702 radiomic features were obtained from two regions of interest (ROI) on both contrast-enhanced lesion, and non-enhancing T2 hyperintense lesion of tumor, overlaid on the CBV map. The overall workflow from image process to analysis is given in Fig. 2, and the obtained radiomic profile of recurrent glioblastoma patients (*n* = 125) is given in Supplementary Fig. 1.

### Performance and interpretation of prediction models for recurrence patterns

**Figure 3:**
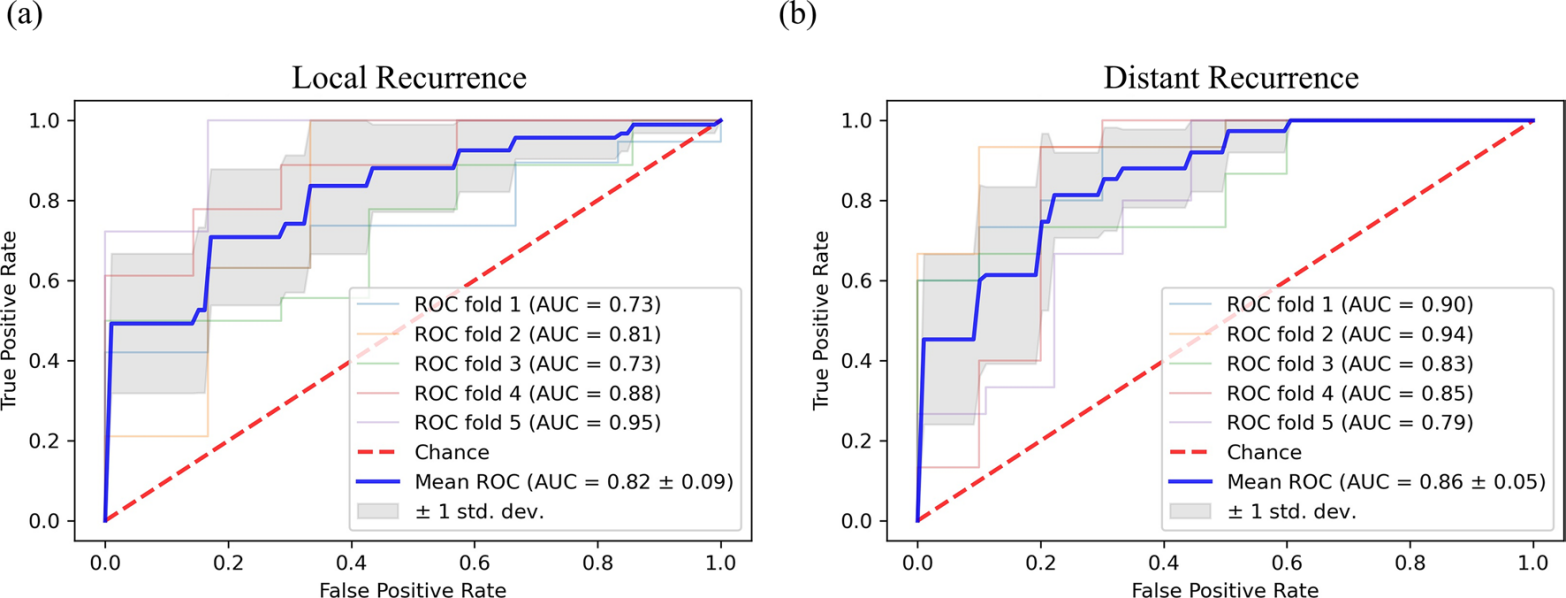
Receiver operating characteristics (ROC) curves of prediction models for recurrence patterns with fivefold cross validation for (**a**) local recurrence, and (**b**) distant recurrence.

The sensitivity, specificity, accuracy, and area under the curve (AUC) of the prediction model for (1) LR vs non-LR was for 94.59%, 100.00%, 97.30%, and 0.995 (0.993–0.996) for discovery set, and 94.44%, 83.33%, 91.67%, and 0.969 (0.903–1.000) for validation set; and (2) DR vs non-DR was for 93.33%, 100.00%, 96.67%, and 0.986 (0.982–0.990) for discovery set, and 93.33%, 80.00%, 88.00%, and 0.864 (0.726–0.976) for validation set, respectively (Table 2). Receiver operating characteristic (ROC) curves for validation set, and training curves for loss and accuracy of each model, are given in Supplementary Fig. 2. In fivefold cross validation, mean AUC was 0.82 ± 0.09 for LR; and 0.86 ± 0.05 for DR (Fig. 3).

**Table 2:** Diagnostic performance of the prediction model for each recurrence pattern: discovery and validation set.

	Discovery set				Validation set			
	Sensitivity (%)	Specificity (%)	Accuracy (%)	AUC (95% CI)	Sensitivity (%)	Specificity (%)	Accuracy (%)	AUC (95% CI)
Local recurrence	94.59	100.00	97.30	0.995 (0.993–0.996)	94.44	83.33	91.67	0.969 (0.903–1.000)
Distant recurrence	93.33	100.00	96.67	0.986 (0.982–0.990)	93.33	80.00	88.00	0.864 (0.726–0.976)

Discovery and validation set was randomly split the total dataset (*n* = 192) into 8:2 ratio.

*AUC* area under the curve, *CI* confidence interval.

**Figure 4:**
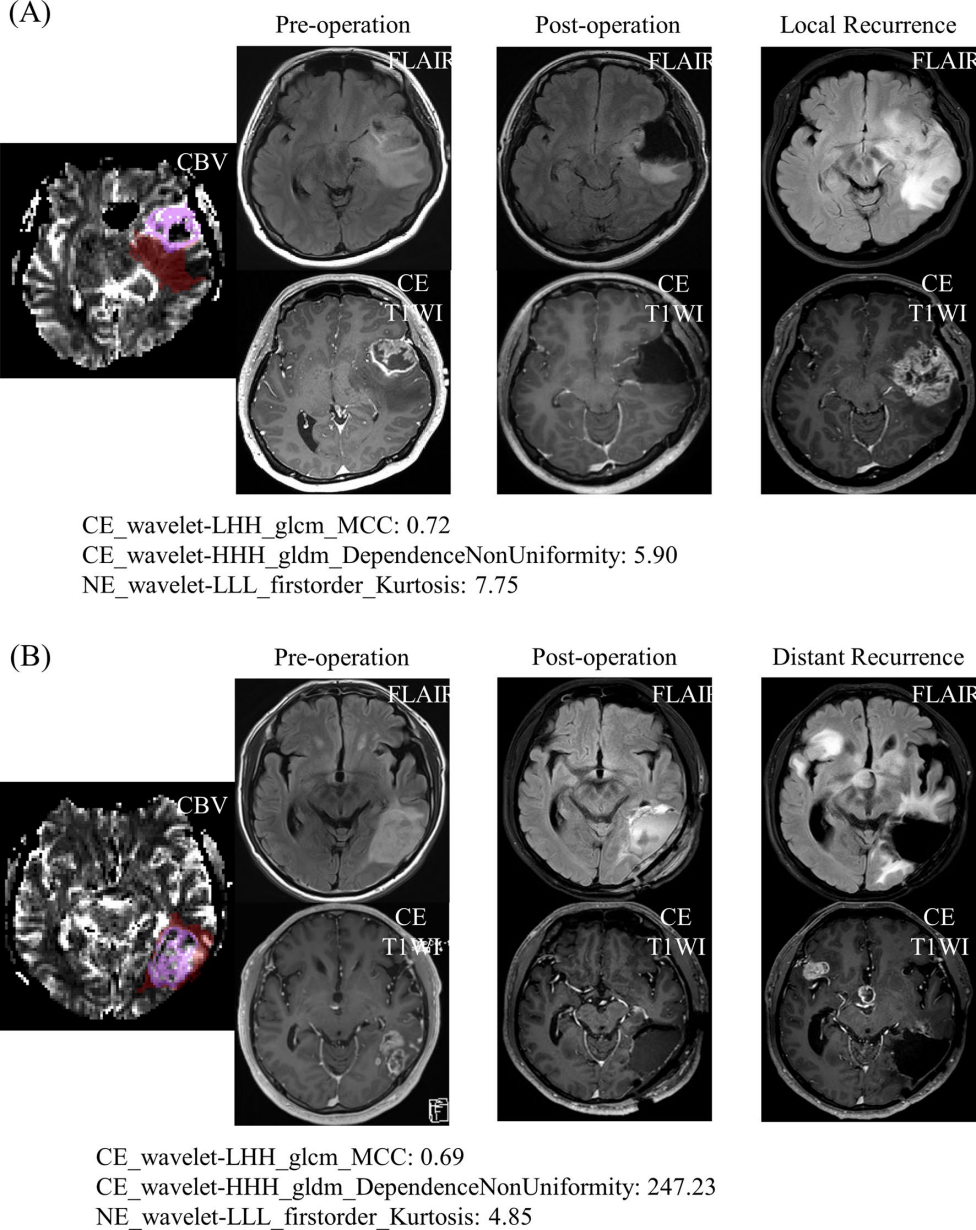
Representative glioblastoma cases with local recurrence (**A**) and with distant recurrence (**B**), respectively. (**A**) A glioblastoma patient who had early local recurrence (recurrence free surival = 12 months) after surgery. The contrast-enhanced glioblastoma with necrosis and high CBV was noted on pre-operative MRI, and total resection of the contrast-enhanced area was performed. In this patient, local recurrence was developed on follow-up MRI. (**B**) A glioblastoma patient who had early distant recurrence (recurrence free surival = 11 months) after surgery. The contrast-enhanced glioblastoma with necrosis and high CBV was noted on pre-operative MRI, and total resection of the contrast-enhanced area was performed. In this patient, distant recurrence was developed in the right sylvian fissure and suprasellar area. The distant recurrence case had a 42 times larger value of *CE_wavelet_HHH_gldm_DependenceNonUniformity* of CBV map, which represents the non-uniformity of gray level values, and thus heterogeneity of tumor, compared with the local recurrence case. Tumor ROI mask is overlaid on rCBV map (leftmost): contrast-enhanced tumor (CE) (purple), and nonenhanicng T2 hyperintense lesion (NE) (brown).

For each model, force plots, decision plots, and summary plots^15^ (detailed in Supplementary material), were obtained from Shapley additive explanations (SHAP) (given in Supplementary Fig. 3), and top 10 important features with largest feature importance values are listed in the Table 3. Full list of 32 features of prediction model for local and distant recurrence are given in Supplementary Tables 2 and 3, respectively. The representative cases with LR and DR are given in Fig. 4.

**Table 3:** Top 10 important features of neural network models to predict each recurrence pattern.

Recurrence pattern	Feature names^†^	Importance values
Local recurrence (LR vs. non-LR group)	NE_wavelet_LLL_firstorder_10Percentile	14.48
	NE_wavelet_LLL_firstorder_Kurtosis*	9.00
	NE_original_shape_LeastAxisLength	7.38
	CE_wavelet_LHH_glcm_ClusterShade	7.18
	NE_original_glszm_LowGrayLevelZoneEmphasis	6.71
	CE_wavelet_HLL_glcm_Idn	6.19
	CE_wavelet_LHH_glcm_MCC*	6.06
	NE_original_glcm_InverseVariance	5.35
	NE_wavelet_HLH_glszm_SizeZoneNonUniformityNor	4.74
	CE_wavelet_HHL_firstorder_InterquartileRange	4.70
Distant recurrence (DR vs. non-DR group)	CE_wavelet_HHH_gldm_DependenceNonUniformity*	5.99
	NE_wavelet_LHH_firstorder_Kurtosis	3.65
	NE_wavelet_HHH_firstorder_Energy	3.43
	CE_wavelet_HLH_firstorder_Maximum	3.09
	NE_wavelet_HLH_firstorder_Skewness	3.02
	NE_wavelet_HLL_glcm_ClusterShade	2.09
	CE_original_glszm_SmallAreaLowGrayLevelEmphasis	1.95
	CE_original_shape_Elongation	1.78
	NE_wavelet_LLH_glcm_Correlation	1.48
	NE_wavelet_LHH_glcm_InverseVariance	1.39

CE, features from T1-weighted contrast-enhanced images; NE, features from non-enhancing T2 high signal intensity area; firstorder, first order features; glcm, gray level co-occurrence matrix features; gldm, gray level dependence matrix features; glszm, gray level size zone matrix features; shape, shape features. The feature was named as region_filter name_feature class_feature name. Feature classes and names can be found in the Supplementary Material.

^†^Important features are listed in descending order of feature importance values.

*Indicates the three selected features for radiomic risk score using Cox-LASSO.

### Radiomic risk score

Using Cox regression with least absolute shrinkage and selection operator (Cox-LASSO), only 3 features had non-zero coefficients and were survived from total 64 features (i.e. 32 features obtained from each of two different prediction model). Next, a radiomic risk score was developed using a linear combination of the 3 selected features with coefficients obtained from Cox-LASSO (Eq. 1), and each patient was stratified into either a high- or low- “radiomic risk group” using the median values of radiomic risk scores for cut-offs^16^.

1 $$ \begin{aligned} Radiomic\;risk\;score & = CE\_wavelet\_LHH\_glcm\_MCC \times 0.219 \\ & \quad + \, CE\_wavelet\_HHH\_gldm\_DependenceNonUniformity \\ & \quad \times \, 0.121 + NE\_wavelet\_LLL\_firstorder\_Kurtosis \\ & \quad \times \,0.068 \\ \end{aligned} $$

**Figure 5:**
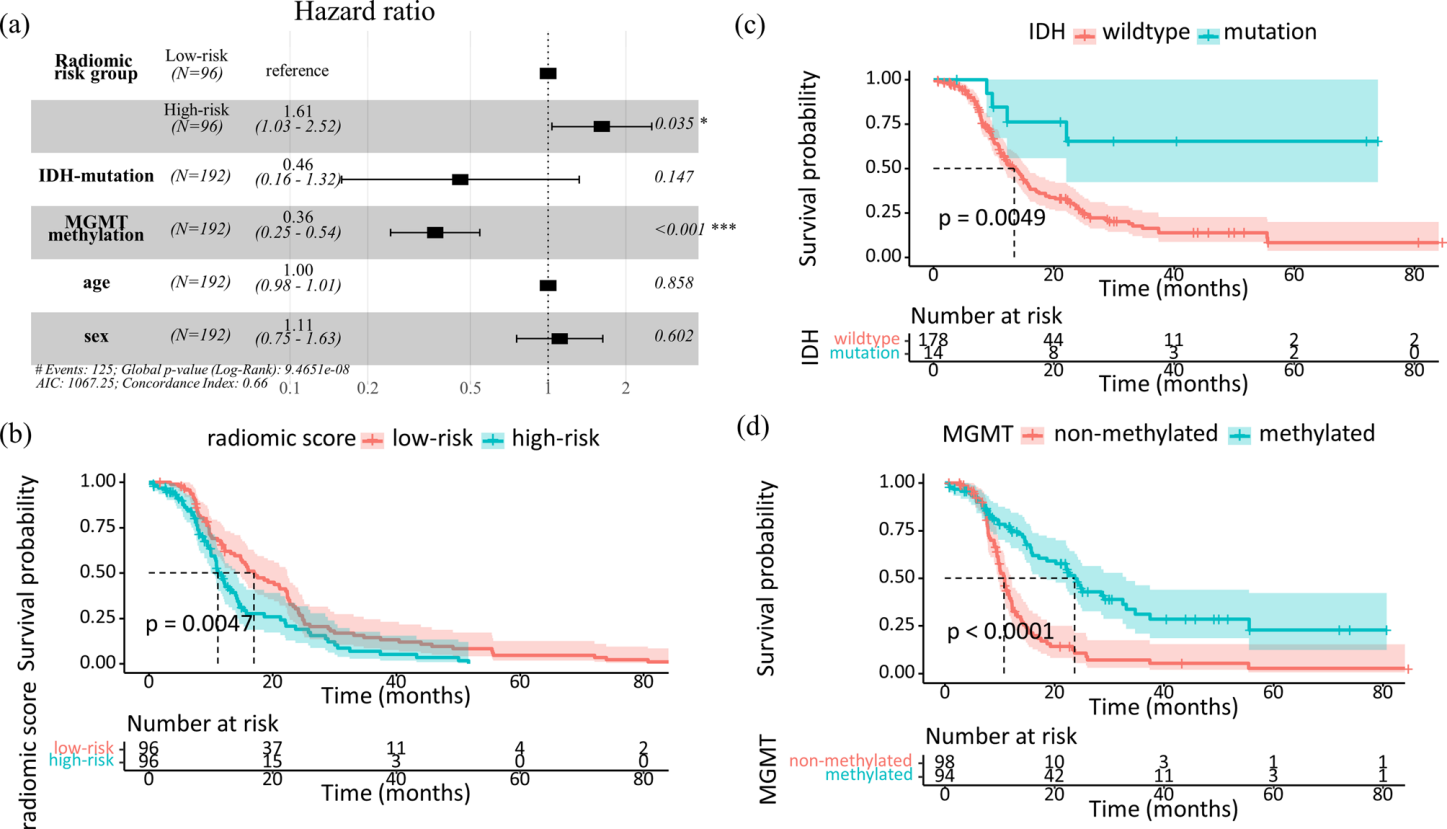
Kaplan–Meier survival curves showing progression free survival (PFS): (**a**) Forest plot of multivariate Cox-regression model; and risk of recurrence was stratified between (**b**) high and low radiomic risk group (*p* = 0.0047), (**c**) *IDH*-mutation and wildtype (*p* = 0.0049), and (**d**) *MGMT*-methylation and unmethylation (*p* < 0.0001). *Note*: *p* values are obtained from the log-rank test which compares two survival functions according to risk group. 95% confidence intervals of survival functions are indicated as gray zone. Bottom tables indicate the actual number of patients at risk for the survival time according to the risk group.

In log-rank test, risk of recurrence was stratified between high and low radiomic risk group (*p* = 0.0047) (Kaplan–Meier plots illustrated in Fig. 5a).

### Multivariate Cox proportional hazard model to predict progression free survival

In multivariate Cox regression model to predict progression free survival (PFS), among clinical and radiomic variables: age, sex, *IDH* status, *MGMT* status, and radiomic risk group, *MGMT* status, and radiomic risk group were significant, and concordance index (C-index) of the multivariate Cox model was 0.66 (standard deviation = 0.03). More specifically, radiomic risk group experienced 1.6 times stronger association toprogression (i.e. hazard ratio (HR) = 1.61; 95% confidence interval (CI), 1.03–2.52; *p* = 0.035). *MGMT*-methylation group experienced about 3 times weaker association to progression (i.e. HR = 0.36; 95% CI, 0.25–0.54; *p* = 6.38 × 10^–7^). A forest plot, showing the variables and HR with CI and *p* values of multivariate Cox-regression model, is given in the Fig. 5a. However, *IDH*-mutation was not significant in the multivariate Cox model (HR = 0.46; 95% CI 0.16–1.32; *p* = 0.147), but significant when *MGMT* status was excepted (HR = 0.26; 95% CI 0.092–0.74; *p* = 0.011) (Supplementary Fig. 4) as well as in log-rank test (*p* = 0.0049). Male sex (HR = 1.11; 95% CI 0.75–1.63; *p* = 0.602), and aged (HR = 1.00; 95% CI 0.98–1.01; *p* = 0.858) were not significant variables. Male sex was not significant when *MGMT* status was excepted as well (HR = 1.01;95% CI 0.72–1.40; *p* = 0.971) (Supplementary Fig. 5). Kaplan–Meier plots for each variable are illustrated in Fig. 5b–d.

## Discussion

The utility of CBV in the prediction of GBM patient outcome has been demonstrated in several previous studies. Other studies have similarly showed that CBV features, associated with tumor aggressiveness, is valuable parameters for prediction of glioma patient survival and prognosis^17–19^. However, single parameter approach has been noted for limited ability in survival prediction^20,21^. Recently, radiomics approach, in combined with building prediction models with large number of parameters such as neural networks has enabled accurate prediction at an individual level^8,22^, though the developed models should be sufficiently validated. Moreover, the individual prediction of the recurrence patterns in GBM is crucial for optimal management of the affected patients^23^. In our study, we evaluated radiomic features from CBV maps to see whether the recurrence patterns in GBM patients, and prognosis could be predicted with these features, using deep learning and Cox-regression, respectively. Specifically, 32 useful radiomic features were selected among 1702 features, and the recurrence pattern of each patient in validation set was predicted using multilayer perceptron network as a classifier, showing excellent performance. Subsequently, “radiomic risk score” was developed using the 3 selected features from the 64 combined radiomic features, which were obtained to predict recurrence patterns, and the score was an independent risk factor of progression in multivariate Cox regression. As a result, the radiomic features of the CBV maps based on non-enhancing T2 hyperintense lesions, and contrast-enhanced tumor, were most helpful for the prediction of the local recurrence and distant recurrence, respectively. Moreover, the PFS could largely be predicted by using the radiomic features of the CBV maps combined with clinical variables.

Despite high performance of the developed prediction model based on radiomics, the data-driven nature of radiomics is insufficient in a way without semantic analysis, because inherently it does not provide additional insight to the biological meanings of selected features^24^. In the present study, we tried semantic analysis to understand the final selected features with high importance values, obtained using SHAP algorithm^15,25^, which catches and provides the “insights” from the developed neural network. In the LR prediction, top three first-order, and shape features extracted from non-enhancing T2 hyperintense lesions, *NE_wavelet_LLL_firstorder_10Percentile*, *NE_wavelet_LLL_firstorder_Kurtosis*, and *NE_original_shape_LeastAxisLength*, showed the highest feature importance values using SHAP^15^, an extendable algorithm for interpretation of neural network models (Table 3): the first two features describe that tumors with higher CBV values (i.e. high *10Percentile*), peaked at higher levels than mean in the histogram of voxel intensity of non-enhancing T2 hyperintense lesions (i.e. high *Kurtosis*), which might reflect higher infiltrating tumor cells in non-enhancing lesions, are associated with LR; and also non-enhancing lesions involving all directions equally (i.e. high *LeastAxisLength)*, surrounding enhancing tumor, are associated with LR, which are consistent with previous studies^26,27^. In summary, high CBV values of nonenhancing T2 hyperintense lesions seemed to be the most useful parameter for LR prediction. Kim et al. also reported that CBV features from nonenhancing regions were useful for anticipating local recurrence, and even more significant if combined with franctional anisotrpy^20^. Our LR prediction model results determined that in the CBV maps, nonenhancing area were more competent than contrast-enhanced regions.

The most important feature of the prediction model for DR was CE T1WI based textural features from CBV maps: *CE_wavelet_HHH_gldm_DependenceNonUniformity* (Table 3). This parameter measures the similarity of dependence through the image, with a higher value indicating more heterogeneity, which might reflect ITH of CBV maps in contrast-enhanced regions. Meanwhile, for both DR, and LR prediction, top important features were mostly wavelet features (Table 3). As the wavelet transform is very useful in texture analysis because of the linearity and the possibility of time/space localizations^28^, recurrence patterns could largely be predicted by textural radiomic features from CBV maps. In addition, texture analysis has proven to be an effective way to measure ITH, as shown in breast cancer, lung cancer, colorectal cancer, etc.^29–31^. In addition, contrast-enhanced areas on CE T1WI could reflect ITH^32^: GBM cells release BBB disrupting factors which are up-regulated with increased malignancy, thus enhancing endothelial cell permeability^32^. Thus, higher signal on CE T1WI implies relatively more compromised BBB in tissue that later recurred, also consistent with infiltrating tumor characteristics^33^. However, the interpretation of the features is only a hypothesis, which requires further validation in cellular level.

DR has the apparent negative impact on survival of patients with malignant gliomas^34^. In addition, the nature of migratory glioma cells is highly associated with matrix adhesion which is mediated by signal transduction cascades used in transmembrane receptors^35^. Various genetic mutations to contributing DR in patients with GBM have been discovered including tumor suppressor gene *PTEN*, gene signaling to block apoptosis *SPOCK1, ANXA11*, and so forth^36^. As a result, DR of GBM is greatly related to highly malignant portions of GBM tumor cells which are well demonstrated in texture features from CBV map based on CE T1WI. In summary, because of DR’s aggressive nature, the feature from contrast-enhanced areas was more important than features from non-enhancing T2 hyperintense lesions in the prediction model for DR, in contrast to LR.

Rapp et al. has reported that recurrence patterns affect the survival in GBM patients^23^. Similarly, in the present study, the radiomic risk variable, or the weighted combination of three selected radiomic features (Eq. 1) to predict the recurrence patterns, could predict the PFS using Cox regression model (C-index, 0.66), when combined with clinical variables: the radiomic risk variable increased the risk (HR = 1.61, *p* = 0.035), and *MGMT* methylation decreased the risk of the recurrence (HR = 0.36, *p* = 3.52 × 10^–4^), which is consistent with the previous study^37^. Interestingly, *IDH* mutation status was not significant (*p* = 0.147) when MGMT status variable was in the multivariate Cox regression model, but significant (*p* = 0.011) when MGMT status was excepted in the Cox regression, which was similar in the previou study^38^. Recently, sex difference has been revealed to be existent in treatment response of glioblastoma^39^. Smits et al. reported that female sex in combined with MGMT methylation—epigenetic silencing for DNA repair of tumor after temozolomide treatment, which has been shown to be more sensitive to chemotherapy^40,41^—showed more favorable outcome to standard therapy^39,42^. However, in our result, sex was not significant prognostic factor when removing MGMT status from the Cox model or not. Two forest plots for two different multivariate Cox models with and without sex variable are given in Supplementary Fig. 4. Adding and removing variables, radiomic risk group and MGMT methylation were the two only consistent significant prognostic factors in several different multivariate Cox regression models.

Regarding radiomic risk group variable, three selected features for radiomic risk variable were *CE_wavelet_LHH_glcm_MCC, CE_wavelet_HHH_gldm_DependenceNonUniformity*, and *NE_wavelet_LLL_firstorder_Kurtosis*. The result that the two features from contrast-enhanced areas is crucial for predicting PFS, was consistent with previous studies that contrast enhancement is strongly associated with poor prognosis^43^. In addition, the two features were texture features regarding *glcm_MCC*, and *gldm_DependenceNonUniformity*, where maximal correlation coefficient (MCC) is a measure of complexity of the texture^44^. Both *MCC* and *DependenceNonUniformity* are also associated with variability of intensity values in contrast-enhanced lesions, which is consistent with that ITH is primarily related with the PFS.

Our study has several limitations. First, due to the retrospective design with relatively small sample size and single-centered data, the generalizability may be limited. Though we reduced the overfitting due to high-dimensionality by using dimension reduction, and data augmentation, the prediction model should be further improved using a larger dataset. Further research with multicentered larger sample size with prospective design should be conducted to validate the generalizability of the developed model. Second, the recurrence is ultimately time-dependent data, however, we specified the recurrence “at 1 year” to treat them as binary outcome to simplify the prediction model. Further improvement can be made integrating time information such as time-to-progression to develop the prediction model. Third, multimodal MRI images were not utilized, and further improvement warranty the potential increase of the model performance. Fourth, semi-automatic segmentation was used to draw the tumor ROIs, which might lower reproducibility and also laborsome. Automatic tumor segmentation algorithm using deep learning could further improve reproducibility and easily applied to large-scale data. Fifth, model comparison using other classifiers such as support vector machine, and random forest was not performed, which might even improve the performance. Sixth, bias field correction was not performed in image preprocessing, which might affect the analysis. Lastly, to date, biological meaning of radiomcs features is not fully understood. Thus, further researches for determining relationships among radiomics data and tumor biology are needed.

Though many different machine learning algorithms, or even a neural network model with large number of parameters, have been utilized for feature selection, and classification in radiomics approach^46–48^, there is no single “ultimate” model that predicts everything^49^. Because the performance of the machine/deep learning model largely depends on the data itself rather than algorithm, which contains sufficient information to predict the specific variable such as prognosis. Moreover, for clinical application of radiomics, reproducibility and repeatability of data should be also validated, and further study is warranted using external validation with different imaging settings such as MR scanner, as well as test–retest analysis, respectively^45^.

In conclusion, the radiomic features from CBV maps based on contrast-enhanced area and non-enhancing T2 hyperintense area were mainly important for predicting DR and LR, respectively. The CBV heterogeneity was a salient parameter for both recurrence patterns as well as survival: LR, DR, and PFS. By using relevant features to predict the recurrence patterns, PFS could largely be regressed/explained in GBM patients using Cox-regression model. Our results might be helpful for the optimal treatment planning as well as clinical trial designs in GBM, avoiding suboptimal patient selection.

## Methods

### Patients

This retrospective study was approved by the Institutional Review Board of Seoul National University Hospital. The Institutional Review Board waived the requirement for informed consent. The study protocol is performed in accordance with the relevant guidelines and regulations. Two hundred and ninety-five consecutive patients (*n* = 295) diagnosed with glioblastoma from April 2010 to September 2019 at Seoul National University Hospital from the radiology report database were enrolled to the study. The followings were the inclusion criteria: patients (a) had a histopathologic diagnosis of GBM for the first time based on the 2016 WHO classification of central nervous system tumors, (b) had preoperative (24–48 h before the operation) and follow-up 3 T MR imaging including contrast enhanced (CE) T1WI, DSC MRI and FLAIR, (c) underwent the standard treatment, concomitant chemoradiotherapy with temozolomide and six cycles of adjuvant temozolomide after maximal (gross-total and near-total; > 95% by volume) surgical resection of contrast-enhanced region, and (d) had follow-up period of ≥ 1 year after surgery. The exclusion criteria were as follows: (a) sub-total resection (< 95% by volume) or biopsy (*n* = 68), (b) inadequate image quality for analysis (*n* = 10), and (c) follow up loss (*n* = 25). Under these inclusion and exclusion criteria, total 192 patients were finally included in our study (Fig. 1). We specified the extent of resection as maximal resection at least (i.e. including complete resection), because we focused to investigate the recurrence pattern when no measurable residual enhancing leseions were left after surgery, excluding the recurrence from measurable residual tumor^26,50^.

All patients routinely visited the outpatient clinic and underwent follow-up brain MR imaging with a brain tumor evaluation protocol at our institution every 3 months for the first 2 years. Then the follow-up period was extended to 6 months if the patient had no evidence of progression, clinically or radiologically. Based on the Response Assessment in Neuro-Oncology (RANO) criteria, the neuro-oncology team of our institution, consisting of radiologists, neurosurgeons, neurooncologists, and radiation oncologists, assessed the progression, and according to the assessment, the patients were classified into disease recurrence and non-recurrence groups. Patients corresponding to any of the followings were considered to have disease recurrence over a 1-year follow-up after the completion of adjuvant temozolomide^51,52^: (a) a greater than 25% increase in the sum of the products of the perpendicular diameters of the enhancing lesions with the smallest tumor measurement, (b) any new lesion, (c) clear clinical deterioration not attributable to causes other than the tumor and (d) clear recurrence of non-measurable disease. Because almost all glioblastoma progresses (i.e. recurs)^53^, and recurrence is time-dependent data in nature, we need an endpoint to assess the type of the recurrence. The median PFS was 335 days in our data, and we evaluated the type of the recurrence at 1 year, which is similar logic to previous clinical trials^54^. The rest of the patients, who are not corresponding to all of the above, were grouped into non-recurrene group. As a result, our study population was categorized into disease recurrence group (*n* = 125), and non-recurrence group (*n* = 67). The progression free survival (PFS) was defined as the interval between the initial diagnosis by MRI examination and the assessment of disease progression, or the last follow-up if the patient had no evidence of disease in the last follow-up^50^.

### Definition of recurrence pattern

Disease recurrence was identified in three types^23^: local recurrence (*n* = 49), distant recurrence (*n* = 32), and combined recurrence (*n* = 44) according to the following criteria. Local recurrence (LR) was defined radiologically as tumor regrowth around the resection cavity, which was considered as non-enhancing T2 hyperintense area after surgery. We defined both subependymal and leptomeningeal seeding as distant recurrence (DR)^55^, because GBM can spread through the CSF along the ventricles, leading to subependymal or ependymal enhancement on MRI. In our study, imaging criteria for subependymal seeding was linear or nodular enhancement of the subependymal region that was remote from the ventricular margin of the primary neoplasm. Leptomeningeal seeding was defined radiologically as linear or nodular enhancement in the subarachnoid space or along the pial surfaces of the brain or spinal cord^56^. While leptomeningeal seeding occurs in 4% of patients with glioma and is thought to be rare, subependymal seeding is associated with higher rate of recurrence of gliomas and the region has long been considered a common site for tumor invasion^57,58^. In addition, despite their seemingly different definition and incidence, Anderson et al.^55^ revealed that subependymal and leptomeningeal seeding have equivalent clinical behavior regarding time to development of the disease, survival time from the diagnosis, rates of hydrocephalus and ventriculoperitoneal shunt placement, etc. Finally, to further analyze features specific to local, and distant recurrence, total enrolled patients (*n* = 192) were grouped into LR (*n* = 93) and non-LR (*n* = 99) group; and DR (*n* = 76) versus non-DR (*n* = 116) group (i.e. binary classification), respectively.

### Imaging protocol

All the MR imaging analyzed in this study was preoperative imaging. For tumor segmentation, the T1W 3D magnetization-prepared rapid acquisition gradient echo sequence before and after administration of gadobutrol (Gadovist; Bayer, Berlin, Germany; at a dose of 0.1 mmol/kg of body weight) and T2-FLAIR imaging were used for the enrolled patients. The DSC MRI protocols were acquired by using dedicated protocols in our institute. MR scan parameters are provided in Supplementary Table 1.

### Image processing and analysis

The MR data including CE T1WI, FLAIR imaging and DSC MRI were transferred from the PACS workstation to a personal computer and processed with a software package (NordicICE v4.1.2; Nordic Neuro Lab, Bergen, Norway).

Prior to drawing regions of interest (ROIs), we performed coregistration of FLAIR and DSC MRI on postcontrast 3D T1WI as structural imaging, which was achieved automatically by an algorithm finding an optimal rigid transformation based on the geometric information^59^. Because 3D MPRAGE was performed for postcontrast T1WI, which is isotropic, T2 FLAIR and DSC MRI was also isotropically resampled (1 mm) to be coregistered to postcontrast T1WI, using trilinear interpolation using FSL (FMRIB Software Library; http://www.fmrib.ox.ac.uk/fsl/)^60^. Manual correction was followed for the best matching nonenhancing T2 highsignal-intensity lesions, if there was geometric distortion due to postsurgical changes. After the motion correction of DSC MRI, deconvolution with the arterial input function was performed in the pharmacokinetic model. Next, a cerebral blood volume (CBV) parametric map calculated from DSC MRI was generated on a pixel-by-pixel basis with leakage correction (detailed in Supplementary Material). Subsequently, two ROIs were drawn slice by slice for tumor volume: (a) enhancing tumor (without necrotic regions) and (b) the non-enhancing T2 hyperintense lesions, which is illustrated in Supplementary Fig. 6. The enhancing tumor areas were drawn semi-automatically on CE T1WI using seed growing and threshold segmentation (and manually, if we need). Then, enhancing tumor areas with necrosis were drawn by adding enhancing tumor and necrosis. Necrosis was defined as areas of relatively hypo-intense regions, frequently located within the enhancing tumoral regions, on CE T1WI^61^. The non-enhancing T2 hyperintense lesions were drawn by subtracting the enhancing tumor areas with necrosis from T2-FLAIR hyperintense lesions^50,52,61–63^. All the images were processed using NordicICE (v4.1.2), and all ROIs were drawn in 3-dimension by three well-trained medical students (J.H.J, K.Y.K and S.W.J), supervised by one expert radiologist (S.H.C., with 17 years of neuro-oncology imaging experience).

Next, the radiomic features from the information contained in the voxels of the segmented structure were extracted using Pyradiomics 2.1.0^64^, a publicly available python package. For reproducible feature extraction, fixed bin size method^65,66^ was used for image intensity discretization, and bin width was chosen to be 5. The radiomic features are composed of seven feature groups: 18 first-order features, 14 shape features (3D), 24 Gy-level co-occurrence matrix (GLCM) features, 16 Gy-level size zone matrix (GLSZM) features, 16 Gy-level run-length matrix (GLRLM) features, 5 neighboring gray tone difference matrix (NGTDM) features, and 14 Gy-level dependence matrix (GLDM) features. The full mathematical description and detailed number of each feature is in Supplementary Material. The original features were radiomics freatures extracted from original images, resulting in 107 features in total.

Wavelet transformation, or filter, was applied to the original input image to extract intensity and textural features from different frequency bands and to obtain fused texture characteristics from two imaging modalities. As a result, each MRI sequence input produced a total of eight wavelet decomposition images as HHH, HLH, HHL, HLL, LHH, LLH, LHL, and LLL images, where ‘H’ stands for a high-pass filter and ‘L’ for a low-pass filter^44^. The first-order features and gray-scale variation features (GLCM, GLRLM, GLSZM, NGTDM, GLDM features) were then applied to the wavelet-transformed images for 18 first-order features + 75 Gy-scale variation features multiplied by eight images, thereby yielding 744 wavelet features. Finally, 851 radiomic features (107 original features, and 744 wavelet features) were respectively extracted from (a) contrast enhancing (CE) lesions and (b) the non-enhancing T2 hyperintense lesions (NE) based on CBV maps, resulting in total 1702 features from each patient. The feature was named as region_filter name_feature class_feature name. Feature classes and feature names are detailed in the Supplementary Material. The overall workflow from image process to analysis is illustrated in Fig. 2, and the radiomic profile of recurrent glioblastoma patients (*n* = 125) is given in the Supplementary Fig. 1.

### Tissue diagnosis and genetic analysis are given in the supplementary materal neural network model

All 1702 radiomic features were z-normalized. Since the radiomic data is a high-dimensional data, or large dimension of feature (*d* = 1702) compared to the sample size (*n* = 192) for the model to be trained, feature selection was essential to avoid “curse of dimensionality”. For feature selection, recursive feature elimination with support vector machine (SVM-RFE)^67^ was performed to select important features by repetitively removing subsets of features with small weights until 32 features were left. Because the selected features, or radiomic data with reduced dimension, will be fed into following neural network model to predict the type of recurrence (Fig. 2), the number of selected features, 32, or a power of 2, was chosen heuristically to be appropriate number considering the size and dimensionality of dataset, which is also a commonly used number for the number of the nodes in hidden layers, and the number of the filters, in neural network models. Using the selected features, we developed the two different neural network models to predict recurrence patterns. Input features (*d* = 32), reduced dimension using SVM-RFE, were passed through 5 hidden layers to get the final output of prediction score for two different binary classification tasks: 1) LR vs non-LR, and 2) DR vs non-DR. Overall model architecture is illustrated in Supplementary Fig. 7.

For evaluation, we performed internal validation using fivefold cross-validation to alleviate the limitation small-sized dataset, reporting more generalized performance. Moreover, to augment the insufficient train/discovery dataset, as well as to deal with class imbalance, we used synthetic minority oversampling technique (SMOTE)^68^, which oversamples the minority class to match the number of samples in majority class, improving the model performance. We only augmented the train/discovery dataset, not validation set, because validation with synthetic data is not valid evaluation. The recurrence prediction model was developed on a randomly split 80% (train/discovery set), and validated on the remaining 20% of data (validation set). To evaluate the diagnostic performance of the model, sensitivity, specificity, and accuracy was obtained, and area under the curve (AUC) was also obtained using receiver operating characteristic (ROC) analysis. To report more generalized performance, fivefold cross validation was also performed to obtain mean $\pm$ standard deviation of AUC for each of the two neural network models for binary classification: LR and DR.

To interpret and understand the features that the neural network model “thinks” are important, we used Shapley additive explanations (SHAP) for each model, which is an additive interpreting model for existing deep learning models^15^. All the implementation is detailed in Supplementary Material.

### Statistical analysis

Subsequent analysis was performed by using software R version 3.6.1 (R Core Team, R Foundation for Statistical Computing, Vienna, Austria)^69^. For all analyses, only *p* < 0.05 was considered statistically significant. Clinical characteristics, including age, sex, date of surgery, radiation dose, date of recurrence, and genetic information, were recorded for each patient. Fisher’s exact test was performed for categorical data. The data for each parameter were assessed for normality with the Kolmogorov–Smirnov test. An unpaired Student’s t test was performed to compare data between the disease recurrence and non-recurrence groups. To examine whether the obtained features predicting recurrence patterns can also predict progression free survival (PFS), we developed a Cox proportional hazard model to regress the PFS with the total 64 selected features obtained above in the prediction model of LR vs non-LR, and DR vs non-DR (32 features for each, respectively). Among 64 features, Cox regression with least absolute shrinkage and selection operator (Cox-LASSO) was performed to select important features (i.e. features with non-zero weights above threshold after LASSO regularization). We optimized the lambda, a hyperparameter for regularization of coefficients in LASSO, using cross-validation, performed by *cv.glmnet* function in *glmnet*^70^, R package. Specifically, tenfold cross-validation was performed to obtain the lambda, which outputs the minimum criterion for training set as default settings. Next, a radiomic score was developed using a linear combination of the selected features with coefficients obtained from Cox-LASSO model (Eq. 1), and each patient was stratified into either a high- or low-“radiomic risk group” using the median values of radiomic scores for cut-offs^16^. Finally, using 5 variables (i.e. 4 clinical variables: sex, age, *MGMT* methylation, *IDH* mutation; and radiomic risk group), a multivariate Cox regression model was developed, and concordance index (C-index) was also obtained to evaluate the performance of the Cox model. All the survival analysis was performed using “survival” R-package^71^.

## Supplementary Information

Supplementary Information

## Data Availability

All relevant data are available on request to correspondence. All codes for model implementation and analysis will soon to be uploaded at https://github.com/kyuchoi.

## References

[1] Wen PY, Kesari S (2008). Malignant gliomas in adults. N. Engl. J. Med..

[2] Ekinci G (2003). Early-postoperative magnetic resonance imaging in glial tumors: prediction of tumor regrowth and recurrence. Eur. J. Radiol..

[3] Sarkaria JN (2018). Is the blood–brain barrier really disrupted in all glioblastomas? A critical assessment of existing clinical data. Neuro Oncol..

[4] Watanabe M, Tanaka R, Takeda N (1992). Magnetic resonance imaging and histopathology of cerebral gliomas. Neuroradiology.

[5] Qazi MA (2017). Intratumoral heterogeneity: pathways to treatment resistance and relapse in human glioblastoma. Ann. Oncol. Off. J. Eur. Soc. Med. Oncol..

[6] McGarry SD (2016). Magnetic resonance imaging-based radiomic profiles predict patient prognosis in newly diagnosed glioblastoma before therapy. Tomography (Ann Arbor, Mich.).

[7] Aghi M (2005). Magnetic resonance imaging characteristics predict epidermal growth factor receptor amplification status in glioblastoma. Clin. Cancer Res. Off. J. Am. Assoc. Cancer Res..

[8] Choi KS, Choi SH, Jeong B (2019). Prediction of IDH genotype in gliomas with dynamic susceptibility contrast perfusion MR imaging using an explainable recurrent neural network. Neuro Oncol..

[9] Hajianfar G (2019). Noninvasive O6 methylguanine-DNA methyltransferase status prediction in glioblastoma multiforme cancer using magnetic resonance imaging radiomics features: univariate and multivariate radiogenomics analysis. World Neurosurg..

[10] Kotrotsou A, Zinn PO, Colen RR (2016). Radiomics in brain tumors: an emerging technique for characterization of tumor environment. Magn. Reson. Imaging Clin..

[11] Lee J (2016). Texture feature ratios from relative CBV maps of perfusion MRI are associated with patient survival in glioblastoma. AJNR Am. J. Neuroradiol..

[12] Stummer W, Kamp MA (2009). The importance of surgical resection in malignant glioma. Curr. Opin. Neurol..

[13] van Linde ME (2017). Treatment outcome of patients with recurrent glioblastoma multiforme: a retrospective multicenter analysis. J. Neuro-oncol..

[14] Kim J (2015). Spatiotemporal evolution of the primary glioblastoma genome. Cancer Cell.

[15] Lundberg, S. M. & Lee, S.-I. In *Advances in neural information processing systems.* 4765–4774.

[16] Liang C (2016). The development and validation of a CT-based radiomics signature for the preoperative discrimination of stage I–II and stage III–IV colorectal cancer. Oncotarget.

[17] Boxerman JL, Schmainda KM, Weisskoff RM (2006). Relative cerebral blood volume maps corrected for contrast agent extravasation significantly correlate with glioma tumor grade, whereas uncorrected maps do not. AJNR Am. J. Neuroradiol..

[18] Larsson C (2020). Prediction of survival and progression in glioblastoma patients using temporal perfusion changes during radiochemotherapy. Magn. Reson. Imaging.

[19] Zhang J (2017). Clinical applications of contrast-enhanced perfusion MRI techniques in gliomas: recent advances and current challenges. Contrast Media Mol. Imaging.

[20] Kim JY (2019). Radiomics in peritumoral non-enhancing regions: fractional anisotropy and cerebral blood volume improve prediction of local progression and overall survival in patients with glioblastoma. Neuroradiology.

[21] Stecco A (2011). DTI and PWI analysis of peri-enhancing tumoral brain tissue in patients treated for glioblastoma. J. Neuro-oncol..

[22] Yan J-L (2020). A neural network approach to identify the peritumoral invasive areas in glioblastoma patients by using MR Radiomics. Sci. Rep..

[23] Rapp M (2017). Recurrence pattern analysis of primary glioblastoma. World Neurosurg..

[24] Tomaszewski MR, Gillies RJJR (2021). The biological meaning of radiomic features. Radiology.

[25] Lundberg SM (2018). Explainable machine-learning predictions for the prevention of hypoxaemia during surgery. Nat. Biomed. Eng..

[26] Kim R (2017). Prognosis prediction of non-enhancing T2 high signal intensity lesions in glioblastoma patients after standard treatment: application of dynamic contrast-enhanced MR imaging. Eur. Radiol..

[27] Ruiz-Ontañon P (2013). Cellular plasticity confers migratory and invasive advantages to a population of glioblastoma-initiating cells that infiltrate peritumoral tissue. Stem Cells.

[28] Materka, A. & Strzelecki, M. Texture analysis methods–a review. *Technical university of lodz, institute of electronics, COST B11 report, Brussels*, 9–11 (1998).

[29] Kim J-H (2017). Breast cancer heterogeneity: MR imaging texture analysis and survival outcomes. Radiology.

[30] Yoon SH (2016). Tumor heterogeneity in lung cancer: assessment with dynamic contrast-enhanced MR imaging. Radiology.

[31] Ng F, Kozarski R, Ganeshan B, Goh V (2013). Assessment of tumor heterogeneity by CT texture analysis: Can the largest cross-sectional area be used as an alternative to whole tumor analysis?. Eur. J. Radiol..

[32] Schneider SW (2004). Glioblastoma cells release factors that disrupt blood–brain barrier features. Acta Neuropathol..

[33] Hynes RO (1992). Integrins: versatility, modulation, and signaling in cell adhesion. Cell.

[34] Parsa AT (2005). Prognostic significance of intracranial dissemination of glioblastoma multiforme in adults. J. Neurosurg..

[35] Giese A, Bjerkvig R, Berens ME, Westphal M (2003). Cost of migration: invasion of malignant gliomas and implications for treatment. J. Clin. Oncol. Off. J. Am. Soc. Clin. Oncol..

[36] Kato H (2004). PTEN gene mutation and high MIB-1 labeling index may contribute to dissemination in patients with glioblastoma. J. Clin. Neurosci. Off. J. Neurosurg. Soc. Aust..

[37] Binabaj MM (2018). The prognostic value of MGMT promoter methylation in glioblastoma: a meta-analysis of clinical trials. J. Cell. Physiol..

[38] Qiu J (2021). Transcriptome analysis and prognostic model construction based on splicing profiling in glioblastoma. Oncol. Lett..

[39] Yang W (2019). Sex differences in GBM revealed by analysis of patient imaging, transcriptome, and survival data. Sci. Transl. Med..

[40] Taylor JW, Schiff D (2015). Treatment considerations for MGMT-unmethylated glioblastoma. Curr. Neurol. Neurosci. Rep..

[41] Fukushima T, Takeshima H, Kataoka H (2009). Anti-glioma therapy with temozolomide and status of the DNA-repair gene MGMT. Anticancer Res..

[42] Smits A (2021). Sex disparities in MGMT promoter methylation and survival in glioblastoma: further evidence from clinical cohorts. J. Clin. Med..

[43] Gutman DA (2013). MR imaging predictors of molecular profile and survival: multi-institutional study of the TCGA glioblastoma data set. Radiology.

[44] Wang JZ (2001). Wavelets and imaging informatics: a review of the literature. J. Biomed. Inform..

[45] Shiri I (2020). Repeatability of radiomic features in magnetic resonance imaging of glioblastoma: test–retest and image registration analyses. Med. Phys..

[46] Nazari M, Shiri I, Zaidi H (2021). Radiomics-based machine learning model to predict risk of death within 5-years in clear cell renal cell carcinoma patients. Comput. Biol. Med..

[47] Leger S (2017). A comparative study of machine learning methods for time-to-event survival data for radiomics risk modelling. Sci. Rep..

[48] Nazari M (2020). Noninvasive Fuhrman grading of clear cell renal cell carcinoma using computed tomography radiomic features and machine learning. La Radiol. Med..

[49] Shiri I (2020). Next-generation radiogenomics sequencing for prediction of EGFR and KRAS mutation status in NSCLC patients using multimodal imaging and machine learning algorithms. Mol. Imaging Biol..

[50] Hwang I (2020). Dynamic contrast-enhanced MR imaging of nonenhancing T2 high-signal-intensity lesions in baseline and posttreatment glioblastoma: temporal change and prognostic value. Am. J. Neuroradiol..

[51] Wen PY (2010). Updated response assessment criteria for high-grade gliomas: response assessment in neuro-oncology working group. J. Clin. Oncol. Off. J. Am. Soc. Clin. Oncol..

[52] Yoo R-E (2017). Dynamic contrast-enhanced MR imaging in predicting progression of enhancing lesions persisting after standard treatment in glioblastoma patients: a prospective study. Eur. Radiol..

[53] Seystahl K, Wick W, Weller M (2016). Therapeutic options in recurrent glioblastoma—an update. Crit. Rev. Oncol. Hematol..

[54] Ballman KV (2007). The relationship between six-month progression-free survival and 12-month overall survival end points for phase II trials in patients with glioblastoma multiforme. Neuro Oncol..

[55] Andersen BM, Miranda C, Hatzoglou V, DeAngelis LM, Miller AM (2019). Leptomeningeal metastases in glioma: The Memorial Sloan Kettering cancer center experience. Neurology.

[56] Witham TF (1999). Survival of patients with high grade glioma treated with intrathecal thiotriethylenephosphoramide for ependymal or leptomeningeal gliomatosis. Cancer Interdiscip. Int. J. Am. Cancer Soc..

[57] Waqas M, Iftikhar M, Siddiqui UT, Enam SA (2017). Ependymal enhancement on magnetic resonance imaging for the identification of high-grade gliomas. Surg. Neurol. Int..

[58] Dardis C, Milton K, Ashby L, Shapiro W (2014). Leptomeningeal metastases in high-grade adult glioma: development, diagnosis, management, and outcomes in a series of 34 patients. Front. Neurol..

[59] Pluim JP, Maintz JA, Viergever MA (2003). Mutual-information-based registration of medical images: a survey. IEEE Trans. Med. Imaging.

[60] Jenkinson M, Bannister P, Brady M, Smith SJN (2002). Improved optimization for the robust and accurate linear registration and motion correction of brain images. Neuroimage.

[61] Prasanna P, Patel J, Partovi S, Madabhushi A, Tiwari P (2017). Radiomic features from the peritumoral brain parenchyma on treatment-naïve multi-parametric MR imaging predict long versus short-term survival in glioblastoma multiforme: preliminary findings. Eur. Radiol..

[62] Song YS (2013). True progression versus pseudoprogression in the treatment of glioblastomas: a comparison study of normalized cerebral blood volume and apparent diffusion coefficient by histogram analysis. Korean J. Radiol..

[63] Yoo R-E (2015). Independent poor prognostic factors for true progression after radiation therapy and concomitant temozolomide in patients with glioblastoma: subependymal enhancement and low ADC value. Am. J. Neuroradiol..

[64] Van Griethuysen JJ (2017). Computational radiomics system to decode the radiographic phenotype. Cancer Res..

[65] Carré A (2020). Standardization of brain MR images across machines and protocols: bridging the gap for MRI-based radiomics. Sci. Rep..

[66] Duron L (2019). Gray-level discretization impacts reproducible MRI radiomics texture features. PLoS ONE.

[67] Guyon I, Weston J, Barnhill S, Vapnik V (2002). Gene selection for cancer classification using support vector machines. Mach. Learn..

[68] Chawla NV, Bowyer KW, Hall LO, Kegelmeyer WP (2002). SMOTE: synthetic minority over-sampling technique. J. Artif. Intell. Res..

[69] Team, R. C. R: A language and environment for statistical computing. (2013).

[70] Friedman J, Hastie T, Tibshirani RJ (2010). Regularization paths for generalized linear models via coordinate descent. J Stat Softw.

[71] Therneau, T. M. in *Proceedings of the First Seattle symposium in biostatistics.* 51–84 (Springer).

