# Supplementary material for: Radiomics-based neural network predicts recurrence patterns in glioblastoma using dynamic susceptibility contrast-enhanced MRI
Source: Sci Rep. 2021 May 11;11:9974. doi: 10.1038/s41598-021-89218-z (PMC8113258; doi:10.1038/s41598-021-89218-z)
Supplement: Supplementary file 1 — Supplementary Information [file 41598_2021_89218_MOESM1_ESM.docx]

**Radiomics-based Neural Network Predicts Recurrence Patterns in Glioblastoma Using Dynamic Susceptibility Contrast-Enhanced MRI**

Ka Young Shim^1*^, Sung Won Chung^1*^, Jae Hak Jeong^1*^, Inpyeong Hwang^2^, Chul-Kee Park^3^,

Tae Min Kim^4^, Sung-Hye Park^5^, Jae Kyung Won^5^, Joo Ho Lee^6^, Soon-Tae Lee^7^,

Roh-Eul Yoo^2^, Koung Mi Kang^2^, Tae Jin Yun^2^, Ji-Hoon Kim^2^, and Chul-Ho Sohn^2^,

Kyu Sung Choi^2†^, Seung Hong Choi^1, 2, 8†^

^*^ These three authors equally contributed to this work.

^†^ Co-correspondence

1. Seoul National University College of Medicine, Seoul, Republic of Korea
2. Department of Radiology, Seoul National University Hospital, Seoul, Republic of Korea
3. Department of Neurosurgery and Biomedical Research Institute, Seoul National University Hospital, Seoul, Korea
4. Department of Internal Medicine and Cancer Research Institute, Seoul National University Hospital, Seoul, Korea
5. Department of Pathology, Seoul National University Hospital, Seoul, Korea
6. Department of Radiation Oncology and Cancer Research Institute, Seoul National University Hospital, Seoul, Korea
7. Department of Neurology, Seoul National University Hospital, Seoul, Korea
8. Center for Nanoparticle Research, Institute for Basic Science (IBS), Seoul, Republic of Korea

Keywords: Glioblastoma; Neural Network; Perfusion Weighted MRI; Recurrence; Radiomics

Submission Type: Original Article

**Correspondence**:

Seung Hong Choi, M.D., Ph.D. (main)

Department of Radiology, Seoul National University Hospital,

Seoul, Republic of Korea

101 Daehangno, Jongno-gu, Seoul, 110-744, Republic of Korea

Tel: 82-2-2072-2584; Fax: 82-2-747-7418

E-mail: verocay@snuh.org

Kyu Sung Choi, M.D.

Department of Radiology, Seoul National University Hospital,

Seoul, Republic of Korea

101 Daehangno, Jongno-gu, Seoul, 110-744, Republic of Korea

Tel: 82-10-5042-7247

E-mail: kyuchoi86@gmail.com

Statistical Analysis conducted by Dr. Kyu Sung Choi, MD, Department of Radiology, Seoul National University Hospital, Seoul, Republic of Korea.

**Funding**: This study was supported by a grant from the Korea Healthcare technology R&D Projects, Ministry for Health, Welfare & Family Affairs (HI16C1111), by the Brain Research Program through the National Research Foundation of Korea (NRF) funded by the Ministry of Science, ICT & Future Planning (NRF-2016M3C7A1914002), by Basic Science Research Program through the National Research Foundation of Korea (NRF) funded by the Ministry of Science, ICT & Future Planning (NRF-2020R1A2C2008949 and NRF-2020R1A4A1018714), by Creative-Pioneering Researchers Program through Seoul National University (SNU), and by the Institute for Basic Science (IBS-R006-A1).

The authors disclose no conflicts of interest related to this work.

**Authorship**: Study Design, SC, JJ, KS, IH, CKP, TMK, SHP, JKW, KSC, SHC; Data collection, analysis, interpretation, SC, JJ, KS, KSC, JHL, IHK, STL, REY, KMK, TJY, JHK, CHS, SHC; Figures, SC, JJ, KS, KSC, SHC; Manuscript Writing, SC, JJ, KS, KSC, SHC.

All authors revised and approved the final version of the manuscript.

**Supplementary Material**

**Post-processing for DSC MRI**

The MR data from the DSC MRI were processed with a dedicated software package (Nordic ICE v4.1.2; Nordic Neuro Lab, Bergen, Norway). nCBV maps were obtained and applied an established tracer kinetic model for the first-pass data^1^. First, realignment was performed to minimized patient motion during dynamic scanning. The gamma-variate function, which is an approximation of the first-pass response at it would appear in the absence of recirculation, was fitted to the 1/T2* curves to reduce the effects of recirculation. Dynamic curves were mathematically corrected to reduce contrast-agent leakage effects^2,3^. After the elimination of recirculation and leakage of the contrast agent, CBV was computed with numeric integration of the curve. To minimize variances in the CBV in an individual patient, the pixel-based CBV maps were normalized by dividing every CBV value in a specific section by the CBV value in the unaffected white matter^4^.

**Recursive feature elimination with support vector machine**

Because the inputs are too high-dimensional data (*d*=1,702) for the model to be trained with only a small sized train data (*n*=192), the input features were reduced into 32 from 1,702 using recursive feature elimination with support vector machine (SVM-RFE) as a classifier^5^, implemented in scikit-learn, a Python library. More specifically, the classifier is trained on the initial set of features, computing the ranking of weights of each feature, and the features with smallest ranking are discarded from the current feature set, recursively to obtain a desired number of features^5^.

**Data augmentation using synthetic minority oversampling technique (SMOTE)**

We augmented train data using a strategy called synthetic minority oversampling technique (SMOTE)^6^, as well as to deal with class imbalance in the dataset. In binary classification task, the larger the difference between the number of data of majority and minority class is, the worse the model performs. Also, the more data we have, the better the model performs. Specifically, SMOTE introduce synthetic examples by taking *k* nearest neighbor minority samples, and select a random point along the line segment between them, or taking linear combination of neighbors, in “feature space” rather than “data space”^6^. In this way, we can augment train data, and balancing the number in each class as well, though we have small dataset for training.

**Neural network architectures**

For prediction models, we developed a multilayer perceptron (MLP) for a shallow neural network models. Input features (*d*=32) with reduced dimension using SVM-RFE, were passed through 5 hidden layers to get the final output of prediction score for two different binary classification tasks: 1) LR vs non-LR, and 3) DR vs non-DR. Each hidden layer is a simple linear layer in Pytorch. One-dimensional batch normalization layer, and rectified linear unit (ReLu) was applied as an activation function at the end of each hidden layer. Dropout was applied to final hidden layer to prevent overfitting^7^. A sigmoid function was applied to the final prediction score for the binary classification. Binary cross-entropy function was used as an objective function. Adaptive moment estimation (Adam)^8^ optimizer was used with an initial learning rate of 0.003, and decayed by gamma of 0.2 every 10 epochs. The size of minibatch was 32, and models were trained over 100 epochs, and saved the best model using early stopping. All the neural network models were implemented using Pytorch 1.5.0, and sklearn 0.22.1 on RTX 2080 Ti graphics processing unit (GPU). Overall model architecture is illustrated in Supplementary Figure 6.

**SHapley Additive exPlanations (SHAP)**

To interpret, and understand the features that the neural network model thinks are important, we used SHapley Additive exPlanations (SHAP)^9,10^ for each model. SHAP assigns each feature a Shapley value as an importance value that represents the effect on the model prediction of including that feature. Specifically, Shapley values are computed as a weighted average of the difference of prediction scores between the two models for all possible combinations: a model trained with that feature present, and another model trained the feature withheld. In other words, Shapley values estimate the feature attribution for the model prediction by calculating how much the feature increases the prediction score, comparing prediction scores of the models with and without the feature^9^. Force plots, showing the impact and direction of the “force” of features that makes the prediction score higher or lower, were obtained. Summary plots, showing the importance values of each feature were obtained from the neural network models, and decision plots, both showing the change of model prediction scores adding-up each feature moving from the bottom to the top of the plot, were also obtained^10^.

**Radiomic features**

*(*[*https://pyradiomics.readthedocs.io/en/latest/features.html#module-radiomics.shape*](https://pyradiomics.readthedocs.io/en/latest/features.html#module-radiomics.shape)*)*

Pyradiomics support to extract various radiomic feature classes, and for all classes, we obtained both original and wavelet features, which can be calculated on an original image and a derived image by applying wavelet filter, respectively. Followings are the list of different feature classes.

1. First-order features

The first-order features described the distribution of voxel intensities within the image region defined by the mask through commonly used and basic metrics.

Let X be a set of 𝑁𝑝 voxels included in the ROI. P(i) refers to the first order histogram with 𝑁𝑔 discrete intensity levels, where 𝑁𝑔 is the number of non-zero bins, equally spaced from 0 with a width defined in the binWidth parameter and p(i) is the normalized first order histogram and equal to $\frac{P(i)}{N_{p}}$. 𝜖 is an arbitrarily small positive number (≈2.2×10−16≈2.2×10−16).

| **Energy** | $\sum_{i=1}^{N_{p}} {(X\left( i \right)+c)}^{2}$ | **Interquartile Range** | $P_{75}{- P}_{25}$ |
| --- | --- | --- | --- |
| **Total energy** | $V_{voxel}\sum_{i=1}^{N_{p}} {(X\left( i \right)+c)}^{2}$ | **Range** | max(X)$-$ min(X) |
| **Entropy** | $-\sum_{i=1}^{N_{g}} p(i)\log_{2} (p\left( i \right)+ \epsilon)$ | **Mean absolute deviation(MAD)** | $\frac{1}{N_{p}}\sum_{i=1}^{N_{p}} \left\vert X\left( i \right)-\bar{X} \right\vert$ |
| **Minimum** | min($X$) | **Robust Mean Absolute Deviation**  **(rMAD)** | $\frac{1}{N_{10-90}}\sum_{i=1}^{N_{10-09}} \left\vert X_{10-90}\left( i \right)-\bar{X}_{10-90} \right\vert$ |
| **10^th^ percentile** | The 10^th^ percentile of $X$ | **Root mean squared(RMS)** | $\sqrt{\frac{1}{N_{p}}\sum_{i=1}^{N_{p}} {(X\left( i \right)+c)}^{2}}$ |
| **90^th^ percentile** | The 90^th^ percentile of $X$ | **Skewness** | $\frac{\frac{1}{N_{p}}\sum_{i=1}^{N_{p}} {(X\left( i \right)-\bar{X})}^{3}}{\left( \sqrt{\frac{1}{N_{p}}\sum_{i=1}^{N_{p}} {(X\left( i \right)-\bar{X})}^{2}} \right)^{3}}$ |
| **Maximum** | max($X$) | **Kurtosis** | $\frac{\frac{1}{N_{p}}\sum_{i=1}^{N_{p}} {(X\left( i \right)-\bar{X})}^{4}}{\left( \frac{1}{N_{p}}\sum_{i=1}^{N_{p}} {(X\left( i \right)-\bar{X})}^{2} \right)^{2}}$ |
| **Mean** | $\frac{1}{N_{p}}\sum_{i=1}^{N_{p}} X\left( i \right)$ | **Variance** | $\frac{1}{N_{p}}\sum_{i=1}^{N_{p}} {(X\left( i \right)-\bar{X})}^{2}$ |
| **Median** | The median gray-level intensity within the ROI. | **Uniformity** | $\sum_{i=1}^{N_{g}} {p(i)}^{2}$ |

1. Shape features

Shape features included descriptors of the three-dimensional size and shape of the ROI. They are independent from the gray level intensity distribution in the ROI and were therefore only calculated on the non-derived image and mask.

Unless otherwise specified, features were derived from the approximated shape defined by the triangle mesh. To build this mesh, vertices (points) were first defined as points halfway on an edge between a voxel included in the ROI and one outside the ROI. By connecting these vertices a mesh of connected triangles was obtained, with each triangle defined by 3 adjacent vertices, which shared each side with exactly one other triangle.

This mesh was generated using a marching cubes algorithm. In this algorithm, a 2x2 cube was moved through the mask space. For each position, the corners of the cube were then marked ‘segmented’ (1) or ‘not segmented’ (0). Treating the corners as specific bits in a binary number, a unique cube-index is obtained (0-255). This index was then used to determine which triangles were present in the cube.

These triangles were defined in such a way, that the normal (obtained from the cross product of vectors describing 2 out of 3 edges) were always oriented in the same direction.

Let $N_{v}$ represent the number of voxels included in the ROI and $N_{f}$ represent the number of faces (triangles) defining the Mesh. V is the volume of the mesh in mm^3^ and A is the surface area of the mesh in mm^2^.

| **Elongation** | $\sqrt{\frac{\lambda_{minor}}{\lambda_{major}}}$ | **Maximum 3D diameter** | The largest pairwise Euclidean distance between tumor surface mesh vertices. |
| --- | --- | --- | --- |
| **Flatness** | $\sqrt{\frac{\lambda_{least}}{\lambda_{major}}}$ | **Mesh volume** | $V_{i}=\frac{O_{a_{i}}\cdot(O_{b_{i}}\times O_{c_{i}})}{6}$  $V=\sum_{i=1}^{N_{f}} V_{i}$ |
| **Least axis length** | $4\sqrt{\lambda_{least}}$ | **Minor axis length** | $4\sqrt{\lambda_{minor}}$ |
| **Major axis length** | 4$\sqrt{\lambda_{major}}$ | **Sphericity** | $\frac{\sqrt[3]{36\pi V^{2}}}{A}$ |
| **Maximum 2D diameter (column)** | The largest pairwise Euclidean distance between tumor surface mesh vertices in the row-column plane. | **Surface area** | $A_{i}=\frac{1}{2}\left\vert a_{i}b_{i}\times a_{i}c_{i} \right\vert$  $A=\sum_{i=1}^{N_{f}} A_{i}$ |
| **Maximum 2D diameter(row)** | The largest pairwise Euclidean distance between tumor surface mesh vertices in the column-slice plane. | **Surface area to volume ratio** | $\frac{A}{V}$ |
| **Maximum 2D diameter(slice)** | The largest pairwise Euclidean distance between tumor surface mesh vertices in the row-slice plane. | **Voxel volume** | $V_{voxel}=\sum_{k=1}^{N_{v}} V_{k}$ |

1. Gray scale variation features

Gray scale variation features widely used in pattern recognition, refer to higher order statistical measures and summarize the local spatial arrangement of intensities.

- 1. Gray level co-occurrence matrix(GLCM) features

A Gray Level Co-occurrence Matrix (GLCM) of size $N_{g}\times N_{g}$ described the second-order joint probability function of an image region constrained by the mask and was defined as

P($i$,$j$|δ,θ). The ${(i, j)}^{th}$ element of this matrix represented the number of times the combination of levels $i$ and $j$ occurred in two pixels in the image, that were separated by a distance of δ pixels along angle θ. The distance δ from the center voxel was defined as the distance according to the infinity norm. For δ=1, this resulted in 2 neighbors for each of 13 angles in 3D (26-connectivity) and for δ=2 a 98-connectivity (49 unique angles).

Let ϵ be an arbitrarily small positive number(≈ 2.2×10−16), $P\left( i, j \right)$ be the co-occurrence matrix for an arbitrary δ and θ, $p\left( i, j \right)$ be the normalized co-occurrence matrix and equal to $\frac{P\left( i, j \right)}{\sum P\left( i, j \right)}$, $N_{g}$ be the number of discrete intensity levels in the image, $p_{x}\left( i \right)=\sum_{j=1}^{N_{g}} P\left( i, j \right)$  be the marginal row probabilities, $p_{x}\left( i \right)=\sum_{j=1}^{N_{g}} P\left( i, j \right)$ be the marginal column probabilities, $\mu_{x}$ be the mean gray level intensity of  $p_{x}$and defined as $\mu_{x}=\sum_{i=1}^{N_{s}} p_{x}\left( i \right)i$, $\mu_{y}$ be the mean gray level intensity of  $p_{y}$and defined as $\mu_{y}=\sum_{j=1}^{N_{s}} p_{y}\left( j \right)j$, $\sigma_{x}$ be the standard deviation of $p_{x}$, $\sigma_{y}$ be the standard deviation of $p_{y}$, $p_{x+y}\left( k \right)=\sum_{i=1}^{N_{g}} \sum_{j=1}^{N_{g}} p\left( i,j \right)$, where $i+j=k, and k=2,3,\cdots,2N_{g}$, $p_{x-y}\left( k \right)=\sum_{i=1}^{N_{g}} \sum_{j=1}^{N_{g}} p\left( i,j \right)$, where $\left| i-j \right|=k,$and $k=0,1,\cdots,N_{g}-1$, HX = $-\sum_{i=1}^{N_{g}} p_{x}(i)\log_{2} \left( p_{x}\left( i \right)+\epsilon\right)$ be the entropy of $p_{x}$,

HY = $-\sum_{j=1}^{N_{g}} p_{y}(j)\log_{2} \left( p_{y}\left( j \right)+\epsilon\right)$ be the entropy of $p_{y}$,

HXY = $-\sum_{i=1}^{N_{g}} \sum_{j=1}^{N_{g}} p\left( i,j \right)\log_{2} \left( p\left( i,j \right)+\epsilon\right)$ be the entropy of $p\left( i,j \right),$

HXY1 = $-\sum_{i=1}^{N_{g}} \sum_{j=1}^{N_{g}} p\left( i,j \right)\log_{2} \left( {p_{x}\left( i \right)p}_{y}\left( j \right)+\epsilon\right)$, and

HXY2 = $-\sum_{i=1}^{N_{g}} \sum_{j=1}^{N_{g}} {p_{x}\left( i \right)p}_{y}\left( j \right)\log_{2} \left( {p_{x}\left( i \right)p}_{y}\left( j \right)+\epsilon\right)$

| **Autocorrelation** | $\sum_{i=1}^{N_{g}} \sum_{j=1}^{N_{g}} p\left( i,j \right)ij$ | **Joint average** | $\sum_{i=1}^{N_{g}} \sum_{j=1}^{N_{g}} p\left( i,j \right)i$ |
| --- | --- | --- | --- |
| **Cluster prominence** | $\sum_{i=1}^{N_{g}} \sum_{j=1}^{N_{g}} \left( i+j-\mu_{x}-\mu_{y} \right)^{4}p\left( i,j \right)$ | **Inverse difference(ID)** | $\sum_{k=0}^{N_{g}-1} \frac{p_{x-y}\left( k \right)}{1+k}$ |
| **Cluster shade** | $\sum_{i=1}^{N_{g}} \sum_{j=1}^{N_{g}} \left( i+j-\mu_{x}-\mu_{y} \right)^{3}p\left( i,j \right)$ | **Inverse difference normalized(IDN)** | $\sum_{k=0}^{N_{g}-1} \frac{p_{x-y}\left( k \right)}{1+\left( \frac{k}{N_{g}} \right)}$ |
| **Cluster tendency** | $\sum_{i=1}^{N_{g}} \sum_{j=1}^{N_{g}} \left( i+j-\mu_{x}-\mu_{y} \right)^{2}p\left( i,j \right)$ | **Inverse difference moment(IDM)** | $\sum_{k=0}^{N_{g}-1} \frac{p_{x-y}\left( k \right)}{1+k^{2}}$ |
| **Contrast** | $\sum_{i=1}^{N_{g}} \sum_{j=1}^{N_{g}} \left( i-j \right)^{2}p\left( i,j \right)$ | **Inverse difference moment normalized(IDMN)** | $\sum_{k=0}^{N_{g}-1} \frac{p_{x-y}\left( k \right)}{1+\left( \frac{k^{2}}{{N_{g}}^{2}} \right)}$ |
| **Correlation** | $\frac{\sum_{i=1}^{N_{g}} \sum_{j=1}^{N_{g}} p\left( i,j \right)ij-\mu_{x}\mu_{y}}{\sigma_{x}\left( i \right)\sigma_{y}(i)}$ | **Inverse variance** | $\sum_{k=1}^{N_{g}-1} \frac{p_{x-y}\left( k \right)}{k^{2}}$ |
| **Difference average** | $\sum_{k=0}^{N_{g}-1} kp_{x-y}\left( k \right)$ | **Joint energy** | $\sum_{i=1}^{N_{g}} \sum_{j=1}^{N_{g}} \left( p(i,j) \right)^{2}$ |
| **Difference entropy** | $\sum_{k=0}^{N_{g}-1} {p_{x-y}\left( k \right)log}_{2}\left( p_{x-y}\left( k \right)+\epsilon\right)$ | **Joint entropy** | $-\sum_{i=1}^{N_{g}} \sum_{j=1}^{N_{g}} p(i,j)\log_{2} \left( p(i,j)+\epsilon\right)$ |
| **Difference variance** | $\sum_{k=0}^{N_{g}-1} \left( k-DA \right)^{2}p_{x-y}\left( k \right)$ | **Maximum probability** | $max(p\left( i,j \right))$ |
| **Sum of squares** | $\sum_{i=1}^{N_{g}} \sum_{j=1}^{N_{g}} \left( i-\mu_{x} \right)^{2}p(i,j)$ | **Maximal correlation coefficient(MCC)** | $\sqrt{\begin{aligned} second largest \\ eigenvalue of Q \end{aligned}}$  $Q\left( i,j \right)=\sum_{k=0}^{N_{g}} \frac{p\left( i,k \right)p(j,k)}{p_{x}(i)p_{y}(k)}$ |
| **Sum average** | $\sum_{k=2}^{2N_{g}} p_{x+y}\left( k \right)k$ | **Informational measure of correlation(IMC) 1** | $\frac{HXY-HXY1}{max\left\{ HX,HY \right\}}$ |
| **Sum entropy** | $\sum_{k=2}^{2N_{g}} p_{x+y}\left( k \right)\log_{2} p_{x+y}\left( k \right)+\epsilon)$ | **Informational measure of correlation(IMC) 2** | $\sqrt{1-e^{-2(HXY2-HXY)}}$ |

- 1. Gray level size zone matrix(GLSZM) features

A Gray Level Size Zone (GLSZM) quantified gray level zones in an image. A gray level zone was defined as the number of connected voxels that shared the same gray level intensity. A voxel was considered connected if the distance was 1 according to the infinity norm (26-connected region in a 3D, 8-connected region in 2D). In a gray level size zone matrix $p(i,j)$, the ${(i,j)}^{th}$ element equaled the number of zones with gray level $i$ and size $j$ appeared in image. Contrary to GLCM and GLRLM, the GLSZM was rotation independent, with only one matrix calculated for all directions in the ROI.

Let $N_{g}$ be the number of discreet intensity values in the image, $N_{s}$ be the number of discreet zone sizes in the image, $N_{p}$ be the number of voxels in the image, $N_{z}$ be the number of zones in the ROI, which is equal to $\sum_{i=1}^{N_{g}} \sum_{j=1}^{N_{g}} P(i,j)$ and 1$\leq N_{z}\leq N_{p}$, $P\left( i,j \right)$ be the size zone matrix, and $p(i,j)$ be the normalized size zone matrix, defined as $p\left( i,j \right)=\frac{P(i,j)}{N_{z}}$

| **Small area emphasis(SAE)** | $\frac{\sum_{i=1}^{N_{g}} \sum_{j=1}^{N_{s}} \frac{P(i,j)}{j^{2}}}{N_{z}}$ | **Zone variance(ZV)** | $\sum_{i=1}^{N_{g}} \sum_{j=1}^{N_{g}} {p(i,j)\left( j-\mu\right)}^{2},$  $\mu=\sum_{i=1}^{N_{g}} \sum_{j=1}^{N_{g}} p\left( i,j \right)j$ |
| --- | --- | --- | --- |
| **Large area emphasis(LAE)** | $\frac{\sum_{i=1}^{N_{g}} \sum_{j=1}^{N_{s}} P(i,j)j^{2}}{N_{z}}$ | **Zone entropy(ZE)** | $-\sum_{i=1}^{N_{g}} \sum_{j=1}^{N_{s}} p\left( i,j \right)\log_{2}( p\left( i,j \right)+\epsilon$) |
| **Gray level non-uniformity(GLN)** | $\frac{\sum_{i=1}^{N_{g}} \left( \sum_{j=1}^{N_{s}} P(i,j) \right)^{2}}{N_{z}}$ | **Low gray level zone emphasis(LGLZE)** | $\frac{\sum_{i=1}^{N_{g}} \sum_{j=1}^{N_{s}} \frac{P(i,j)}{i^{2}}}{N_{z}}$ |
| **Gray level non-uniformity normalized(GLNN)** | $\frac{\sum_{i=1}^{N_{g}} \left( \sum_{j=1}^{N_{s}} P(i,j) \right)^{2}}{{N_{z}}^{2}}$ | **High gray level zone emphasis(HGLZE)** | $\frac{\sum_{i=1}^{N_{g}} \sum_{j=1}^{N_{s}} P(i,j)i^{2}}{N_{z}}$ |
| **Size-zone non-uniformity(SZN)** | $\frac{\sum_{j=1}^{N_{s}} \left( \sum_{i=1}^{N_{g}} P(i,j) \right)^{2}}{N_{z}}$ | **Small area low gray level emphasis(SALGLE)** | $\frac{\sum_{i=1}^{N_{g}} \sum_{j=1}^{N_{s}} \frac{P(i,j)}{i^{2}j^{2}}}{N_{z}}$ |
| **Size-zone non-uniformity normalized(SZNN)** | $\frac{\sum_{j=1}^{N_{s}} \left( \sum_{i=1}^{N_{g}} P(i,j) \right)^{2}}{{N_{z}}^{2}}$ | **Small area high gray level emphasis(SAHGLE)** | $\frac{\sum_{i=1}^{N_{g}} \sum_{j=1}^{N_{s}} \frac{P(i,j)i^{2}}{j^{2}}}{N_{z}}$ |
| **Zone percentage(ZP)** | $\frac{N_{z}}{N_{p}}$ | **Large area low gray level emphasis(LALGLE)** | $\frac{\sum_{i=1}^{N_{g}} \sum_{j=1}^{N_{s}} \frac{P(i,j)j^{2}}{i^{2}}}{N_{z}}$ |
| **Gray level variance(GLV)** | $\sum_{i=1}^{N_{g}} \sum_{j=1}^{N_{s}} {p\left( i,j \right)\left( i-\mu\right)}^{2},$  $\mu=\sum_{i=1}^{N_{g}} \sum_{j=1}^{N_{s}} p\left( i,j \right)i$ | **Large area high gray level emphasis(LAHGLE)** | $\frac{\sum_{i=1}^{N_{g}} \sum_{j=1}^{N_{s}} P(i,j)i^{2}j^{2}}{N_{z}}$ |

- 1. Gray level run length matrix(GLRLM) features

A Gray Level Run Length Matrix (GLRLM) quantified gray level runs, which were defined as the length in number of pixels, of consecutive pixels that had the same gray level value. In a gray level run length matrix $P(i,j|\theta)$, the ${(i,j)}^{th}$ element described the number of runs with gray level $i$  and length $i$ occurred in the image (ROI) along angle $\theta$.

Let $N_{g}$ be the number of discreet intensity values in the image, $N_{s}$ be the number of discreet zone sizes in the image, $N_{r}$ be the number of discreet run lengths in the image, $N_{p}$ be the number of voxels in the image,$N_{r}\left( \theta\right)$ be the number of runs in the image along angle $\theta$, which is equal to $\sum_{i=1}^{N_{g}} \sum_{j=1}^{N_{g}} P(i,j|\theta)$ and 1$\leq N_{r}\left( \theta\right)\leq N_{p}$, $P\left( i,j|\theta\right)$ be the run length matrix for an arbitrary direction $\theta$, and $p(i,j|\theta)$ be the normalized run length matrix, defined as $p\left( i,j|\theta\right)=\frac{P(i,j|\theta)}{N_{r}\left( \theta\right)}$.

| **Short run emphasis(SRE)** | $\frac{\sum_{i=1}^{N_{g}} \sum_{j=1}^{N_{r}} \frac{P(i,j\vert\theta)}{j^{2}}}{N_{r}\left( \theta\right)}$ | **Run variance(RV)** | $\sum_{i=1}^{N_{g}} \sum_{j=1}^{N_{r}} {p(i,j\vert\theta)\left( j-\mu\right)}^{2},$  $\mu=\sum_{i=1}^{N_{g}} \sum_{j=1}^{N_{r}} p\left( i,j\vert\theta\right)j$ |
| --- | --- | --- | --- |
| **Long run emphasis(LRE)** | $\frac{\sum_{i=1}^{N_{g}} \sum_{j=1}^{N_{r}} P(i,j\vert\theta)j^{2}}{N_{r}\left( \theta\right)}$ | **Run entropy(RE)** | $-\sum_{i=1}^{N_{g}} \sum_{j=1}^{N_{r}} p\left( i,j\vert\theta\right)\log_{2}( p\left( i,j\vert\theta\right)+\epsilon$) |
| **Gray level non-uniformity(GLN)** | $\frac{\sum_{i=1}^{N_{g}} \left( \sum_{j=1}^{N_{r}} P(i,j\vert\theta) \right)^{2}}{N_{r}\left( \theta\right)}$ | **Low gray level run emphasis(LGLRE)** | $\frac{\sum_{i=1}^{N_{g}} \sum_{j=1}^{N_{r}} \frac{P(i,j\vert\theta)}{i^{2}}}{N_{r}\left( \theta\right)}$ |
| **Gray level non-uniformity normalized(GLNN)** | $\frac{\sum_{i=1}^{N_{g}} \left( \sum_{j=1}^{N_{r}} P(i,j\vert\theta) \right)^{2}}{{N_{r}\left( \theta\right)}^{2}}$ | **High gray level run emphasis(HGLRE)** | $\frac{\sum_{i=1}^{N_{g}} \sum_{j=1}^{N_{r}} P(i,j\vert\theta)i^{2}}{N_{r}\left( \theta\right)}$ |
| **Run length non-uniformity(RLN)** | $\frac{\sum_{j=1}^{N_{r}} \left( \sum_{i=1}^{N_{g}} P(i,j\vert\theta) \right)^{2}}{N_{r}\left( \theta\right)}$ | **Short run low gray level emphasis(SRLGLE)** | $\frac{\sum_{i=1}^{N_{g}} \sum_{j=1}^{N_{r}} \frac{P(i,j\vert\theta)}{i^{2}j^{2}}}{N_{r}\left( \theta\right)}$ |
| **Run length non-uniformity normalized(RLNN)** | $\frac{\sum_{j=1}^{N_{r}} \left( \sum_{i=1}^{N_{g}} P(i,j\vert\theta) \right)^{2}}{{N_{r}\left( \theta\right)}^{2}}$ | **Short run high gray level emphasis(SRHGLE)** | $\frac{\sum_{i=1}^{N_{g}} \sum_{j=1}^{N_{r}} \frac{P(i,j\vert\theta)i^{2}}{j^{2}}}{N_{z}}$ |
| **Run percentage(RP)** | $\frac{N_{r}\left( \theta\right)}{N_{p}}$ | **Long run low gray level emphasis(LRLGRE)** | $\frac{\sum_{i=1}^{N_{g}} \sum_{j=1}^{N_{r}} \frac{P(i,j\vert\theta)j^{2}}{i^{2}}}{N_{r}\left( \theta\right)}$ |
| **Gray level variance(GLV)** | $\sum_{i=1}^{N_{g}} \sum_{j=1}^{N_{r}} {p\left( i,j\vert\theta\right)\left( i-\mu\right)}^{2},$  $\mu=\sum_{i=1}^{N_{g}} \sum_{j=1}^{N_{r}} p\left( i,j\vert\theta\right)i$ | **Long run high gray level emphasis(LRHGLE)** | $\frac{\sum_{i=1}^{N_{g}} \sum_{j=1}^{N_{r}} P(i,j\vert\theta)i^{2}j^{2}}{N_{r}\left( \theta\right)}$ |

- 1. Neighboring gray tone difference matrix(NGTDM) features

A Neighboring Gray Tone Difference Matrix(NGTDM) quantified the difference between a gray value and the average gray value of its neighbors within distance δ. The sum of absolute differences for gray level $i$ was stored in the matrix.

Let $X_{gl}$ be a set of segmented voxels and $x_{gl}(j_{x}$, $j_{y}, j_{z})\in X_{gl}$ be the gray level of a voxel at postion $(j_{x}$,$j_{y}, j_{z})$, then the average gray level of the neigborhood is:

$\bar{A}_{i}=\bar{A}(j_{x}$,$j_{y}, j_{z})=\frac{1}{W}\sum_{k_{x}=-\delta}^{\delta} \sum_{k_{y}=-\delta}^{\delta} \sum_{k_{z}=-\delta}^{\delta} x_{gl}(j_{x}+k_{x}, j_{y}+k_{y}, j_{z}+k_{z}),$

where $(k_{x}$,$k, k_{z})\neq(0, 0, 0)$ and $x_{gl}(j_{x}+k_{x}, j_{y}+k_{y}, j_{z}+k_{z})\in X_{gl}$

$W$ is the number of voxels in the neighborhood that are also in $X_{gl}$.

Let $n_{i}$ be the number of voxels in $X_{gl}$ with gray level $i$, $N_{v,p}$ be the total number of voxels in $X_{gl}$ and equal to $\sum n_{i}$(i.e. the number of voxels with a valid region; at least 1 neighbor). $N_{v,p}\leq N_{p}$ , where $N_{p}$ is the total number of voxels in the ROI, $p_{i}$ be the gray level probability and equal to $n_{i}$/$N_{v}$, $s_{i}=\left\{ \begin{matrix} \sum^{n_{i}} \left| i-\bar{A}_{i} \right| & for & n_{i}\neq0 \\ 0 & for & n_{i}=0 \end{matrix} \right.$ be the sum of absolute differences for gray level $i$, $N_{g}$ be the number of discreet gray levels, and $N_{g,p}$be the number of gray levels where $p_{i}\neq0$.

| **Coarseness** | $\frac{1}{\sum_{i=1}^{N_{g}} p_{i}s_{i}}$ | **Complexity** | $\frac{1}{N_{v,p}}\sum_{i=1}^{N_{g}} \sum_{j=1}^{N_{g}} \left\vert i-j \right\vert\frac{p_{i}s_{i}+p_{j}s_{j}}{p_{i}{+p}_{i}}$,  where $p_{i}\neq0, p_{j}\neq0$ |
| --- | --- | --- | --- |
| **Busyness** | $\frac{\sum_{i=1}^{N_{g}} p_{i}s_{i}}{\sum_{i=1}^{N_{g}} \sum_{j=1}^{N_{g}} \left\vert{ip}_{i}-jp_{j} \right\vert}$,  where $p_{i}\neq0, p_{j}\neq0$ | **Strength** | $\frac{\sum_{i=1}^{N_{g}} \sum_{j=1}^{N_{g}} (p_{i}+p_{j}){(i-j)}^{2}}{\sum_{i=1}^{N_{g}} s_{i}}$),  where $p_{i}\neq0, p_{j}\neq0$ |
| **Contrast** | $\left( \frac{1}{N_{g,p}(N_{g,p}-1)}\sum_{i=1}^{N_{g}} \sum_{j=1}^{N_{g}} p_{i}p_{j}{(i-j)}^{2} \right)\left( \frac{1}{N_{v,p}}\sum_{i=1}^{N_{g}} s_{i} \right)$, where $p_{i}\neq0, p_{j}\neq0$ | | |

- 1. Gray level dependence matrix(GLDM) features

A Gray Level Dependence Matrix (GLDM) quantified gray level dependencies in an image. A gray level dependency was defined as the number of connected voxels within distance δ that are dependent on the center voxel. A neighboring voxel with gray level $j$ was considered dependent on center voxel with gray level $i$ if |$i-j$|≤α. In a gray level dependence matrix $P(i,j)$, the  ${(i,j)}^{th}$ element described the number of times a voxel with gray level $i$ with $j$ dependent voxels in its neighborhood appears in image.

Let $N_{g}$ be the number of discreet intensity values in the image, $N_{d}$ be the number of discreet dependency sizes in the image, $N_{z}$ be the number of dependency zones in the image, which is equal to $\sum_{i=1}^{N_{g}} \sum_{j=1}^{N_{d}} P(i,j)$, $P\left( i,j \right)$ be the dependence matrix, and $p(i,j)$ be the normalized dependence matrix, defined as $p\left( i,j \right)=\frac{P(i,j)}{N_{z}}$

| **Small dependence emphasis(SDE)** | $\frac{\sum_{i=1}^{N_{g}} \sum_{j=1}^{N_{d}} \frac{P(i,j)}{i^{2}}}{N_{z}}$ | **Dependence variance(DV)** | $\sum_{i=1}^{N_{g}} \sum_{j=1}^{N_{d}} {p(i,j)\left( j-\mu\right)}^{2},$  $\mu=\sum_{i=1}^{N_{g}} \sum_{j=1}^{N_{d}} p\left( i,j \right)j$ |
| --- | --- | --- | --- |
| **Large dependence emphasis(LDE)** | $\frac{\sum_{i=1}^{N_{g}} \sum_{j=1}^{N_{d}} P(i,j)j^{2}}{N_{z}}$ | **Low gray level emphasis(LGLE)** | $\frac{\sum_{i=1}^{N_{g}} \sum_{j=1}^{N_{d}} \frac{P(i,j)}{i^{2}}}{N_{z}}$ |
| **Gray level non-uniformity(GLN)** | $\frac{\sum_{i=1}^{N_{g}} \left( \sum_{j=1}^{N_{d}} P(i,j) \right)^{2}}{N_{z}}$ | **High gray level emphasis(HGLE)** | $\frac{\sum_{i=1}^{N_{g}} \sum_{j=1}^{N_{d}} P(i,j)i^{2}}{N_{z}}$ |
| **Dependence non-uniformity(DN)** | $\frac{\sum_{j=1}^{N_{d}} \left( \sum_{i=1}^{N_{g}} P(i,j) \right)^{2}}{N_{z}}$ | **Small dependence low gray level emphasis(SDLGLE)** | $\frac{\sum_{i=1}^{N_{g}} \sum_{j=1}^{N_{d}} \frac{P(i,j)}{i^{2}j^{2}}}{N_{z}}$ |
| **Dependence non-uniformity normalized(DNN)** | $\frac{\sum_{j=1}^{N_{d}} \left( \sum_{i=1}^{N_{g}} P(i,j) \right)^{2}}{{N_{z}}^{2}}$ | **Small dependence high gray level emphasis(SDHGLE)** | The joint distribution of small dependence with higher gray-level values. |
| **Dependence entropy(DE)** | $-\sum_{i=1}^{N_{g}} \sum_{j=1}^{N_{d}} p\left( i,j \right)\log_{2}( p\left( i,j \right)+\epsilon$) | **Large dependence high gray level emphasis(LDHGLE)** | $\frac{\sum_{i=1}^{N_{g}} \sum_{j=1}^{N_{d}} P(i,j)i^{2}j^{2}}{N_{z}}$ |
| **Gray level variance(GLV)** | $\sum_{i=1}^{N_{g}} \sum_{j=1}^{N_{d}} {p\left( i,j \right)\left( i-\mu\right)}^{2},$  $\mu=\sum_{i=1}^{N_{g}} \sum_{j=1}^{N_{d}} p\left( i,j \right)i$ | **Large dependence low gray level emphasis(LDLGLE)** | $\frac{\sum_{i=1}^{N_{g}} \sum_{j=1}^{N_{d}} \frac{P(i,j)j^{2}}{i^{2}}}{N_{z}}$ |

**Tissue diagnosis and genetic analysis**

Immunohistochemical staining was performed using a BenchMark XT (automated immunohistochemical slide staining system, Roche Diagnostics, IN, USA). Immunohistochemistry (IHC) was performed on individual whole block sections using antibodies against IDH1 R132H mutant protein (H09, Dianova, Hamburg, Germany, 1:50). If IDH1 IHC was negative, direct Sanger sequencing of IDH1/IDH2 was performed. O6 methylguanine methyltransferase methylation-Specific PCR (MGMT-MSP) using a methylation EZ kit was used to evaluate the methylation status of the MGMT promoter.

**Supplementary Tables**

**Supplementary Table 1.** MRI scan parameters

|  | **Discovery MR750W****(GE)** | **Verio****(Siemens)** | **Skyra** **(Siemens)** |
| --- | --- | --- | --- |
| Field strength (T) | 3.0 | 3.0 | 3.0 |
| Head coil channel | 32 | 32 | 64 |
| T1WI |  |  |  |
| TR(ms) | 8.4 | 1420 | 1600 |
| TE(ms) | 3.2 | 1.9 | 2.8 |
| FA(°) | 12 | 9 | 9 |
| NEX | 1 | 1 | 1 |
| Matrix | 256x230 | 256x232 | 256x248 |
| Section thickness(mm) | 1 | 1 | 1 |
| Intersection gap(mm) | 0 | 0 | 0 |
| FOV(mm) | 256x256 | 249x249 | 249x249 |
| T2WI |  |  |  |
| TR(ms) | 5124.5 | 5100 | 5100 |
| TE(ms) | 92.6 | 89 | 89 |
| FA(°) | 142 | 16 | 150 |
| NEX | 2 | 3 | 3 |
| Matrix | 448x448 | 640x384 | 640x324 |
| Section thickness(mm) | 5 | 5 | 5 |
| Intersection gap(mm) | 1 | 1 | 1 |
| FOV(mm) | 220x220 | 199x220 | 185x220 |
| DSC-MRI |  |  |  |
| TR(ms) | 1500 | 1600 | 1600 |
| TE(ms) | 29.3 | 30 | 30 |
| FA(°) | 60 | 90 | 90 |
| NEX | 1 | 1 | 1 |
| Matrix | 100x100 | 128x128 | 128x128 |
| Section thickness(mm) | 5 | 6 | 6 |
| Intersection gap(mm) | 1.5 | 0.9 | 0.9 |
| FOV(mm) | 240x240 | 240x240 | 240x240 |
| Scan time | 1 min 30 sec | 1 min 44 sec | 1 min 44 sec |

*Abbreviations*: MRI=magnetic resonance imaging; T1WI=T1-weighted imaging; T2WI=T2-weighted imaging; TR=repetition time; TE=echo time; FA=flip angle; NEX=number of excitations; FOV=field of view; DSC-MRI=dynamic susceptibility contrast (DSC) perfusion MR

**Supplementary Table 2.** The list of 32 feature names of prediction model for local recurrence.

| **Feature names** | **Importance values** |
| --- | --- |
| NE_wavelet_LLL_firstorder_10Percentile | 14.48369 |
| NE_wavelet_LLL_firstorder_Kurtosis | 9.001474 |
| NE_original_shape_LeastAxisLength | 7.378004 |
| CE_wavelet_LHH_glcm_ClusterShade | 7.184105 |
| NE_original_glszm_LowGrayLevelZoneEmphasis | 6.705839 |
| CE_wavelet_HLL_glcm_Idn | 6.19124 |
| CE_wavelet_LHH_glcm_MCC | 6.064995 |
| NE_original_glcm_InverseVariance | 5.348557 |
| NE_wavelet_HLH_glszm_SizeZoneNonUniformityNormalized | 4.737542 |
| CE_wavelet_HHL_firstorder_InterquartileRange | 4.704127 |
| CE_wavelet_HHL_glrlm_ShortRunLowGrayLevelEmphasis | 4.138952 |
| CE_wavelet_HLH_firstorder_Median | 3.33872 |
| CE_wavelet_HLH_firstorder_Mean | 3.283767 |
| CE_original_shape_Flatness | 3.131644 |
| CE_original_shape_Elongation | 2.598156 |
| CE_wavelet_HHH_glcm_MCC | 2.332484 |
| NE_wavelet_LLH_glcm_MCC | 2.2288 |
| CE_wavelet_HHH_firstorder_TotalEnergy | 2.188942 |
| CE_wavelet_LLH_glcm_Autocorrelation | 2.086704 |
| NE_wavelet_HLL_glrlm_RunVariance | 2.083786 |
| NE_wavelet_HLH_gldm_LargeDependenceHighGrayLevelEmphasis | 1.710105 |
| NE_wavelet_HHL_firstorder_Mean | 1.590431 |
| CE_wavelet_LHL_glcm_Autocorrelation | 1.477843 |
| CE_wavelet_LLH_glcm_DifferenceEntropy | 1.303521 |
| NE_wavelet_LLH_glcm_Idn | 1.287185 |
| CE_wavelet_LHL_firstorder_Kurtosis | 1.263671 |
| NE_wavelet_LHL_glszm_LargeAreaLowGrayLevelEmphasis | 1.009944 |
| NE_wavelet_HHL_glcm_JointAverage | 0.675962 |
| CE_wavelet_LLL_gldm_LargeDependenceLowGrayLevelEmphasis | 0.675705 |
| CE_wavelet_HLH_glcm_ClusterShade | 0.674702 |
| NE_wavelet_HLL_glcm_ClusterProminence | 0.590948 |
| NE_wavelet_LLH_firstorder_Skewness | 0.336612 |

**Supplementary Table 3.** The list of 32 feature names of prediction model for distant recurrence.

| **Feature names** | **Importance values** |
| --- | --- |
| CE_wavelet_HHH_gldm_DependenceNonUniformity | 5.993539 |
| NE_wavelet_LHH_firstorder_Kurtosis | 3.651738 |
| NE_wavelet_HHH_firstorder_Energy | 3.430936 |
| CE_wavelet_HLH_firstorder_Maximum | 3.09013 |
| NE_wavelet_HLH_firstorder_Skewness | 3.021324 |
| NE_wavelet_HLL_glcm_ClusterShade | 2.090572 |
| CE_original_glszm_SmallAreaLowGrayLevelEmphasis | 1.949529 |
| CE_original_shape_Elongation | 1.78043 |
| NE_wavelet_LLH_glcm_Correlation | 1.479578 |
| NE_wavelet_LHH_glcm_InverseVariance | 1.386424 |
| CE_wavelet_LLH_glcm_Idmn | 1.317762 |
| NE_wavelet_HLL_glcm_Imc2 | 1.295111 |
| NE_wavelet_HHL_glcm_Autocorrelation | 1.282338 |
| NE_original_glcm_MCC | 1.263919 |
| NE_wavelet_LHH_gldm_DependenceVariance | 1.243702 |
| NE_wavelet_LHH_firstorder_Mean | 1.180579 |
| NE_wavelet_HLL_glszm_SizeZoneNonUniformityNormalized | 1.157828 |
| NE_wavelet_LHH_firstorder_Skewness | 1.156759 |
| NE_wavelet_HHL_firstorder_Skewness | 1.079248 |
| NE_wavelet_LLH_gldm_SmallDependenceHighGrayLevelEmphasis | 1.010271 |
| NE_wavelet_LLH_glcm_Imc2 | 0.993843 |
| CE_wavelet_LHH_glrlm_GrayLevelNonUniformityNormalized | 0.846932 |
| CE_wavelet_HHH_ngtdm_Strength | 0.759781 |
| NE_wavelet_LHH_firstorder_Median | 0.737652 |
| CE_wavelet_HLH_firstorder_Skewness | 0.708558 |
| CE_original_ngtdm_Busyness | 0.594453 |
| CE_wavelet_LHL_glcm_ClusterProminence | 0.356374 |
| CE_wavelet_LLH_firstorder_Kurtosis | 0.341247 |
| NE_wavelet_HLL_gldm_LargeDependenceLowGrayLevelEmphasis | 0.29417 |
| CE_wavelet_HLL_glcm_ClusterShade | 0.28559 |
| NE_wavelet_HLH_glcm_ClusterShade | 0.186525 |
| NE_wavelet_HHL_glszm_SmallAreaEmphasis | 0.113826 |

**Supplementary Figures**

**
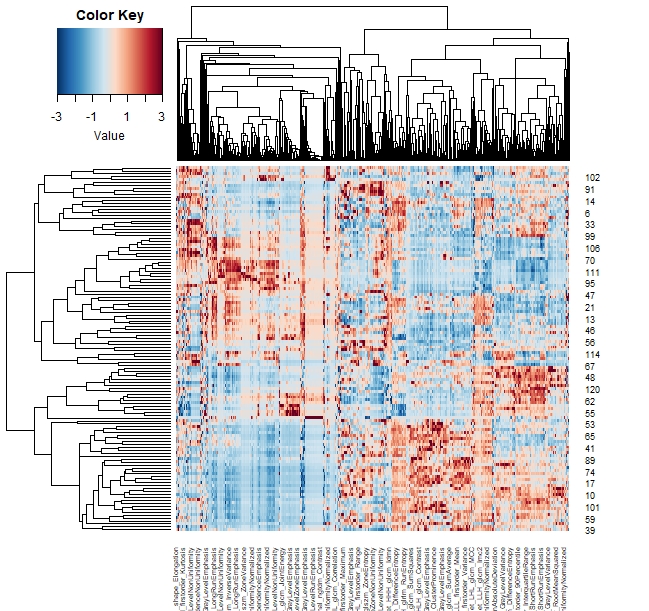
**

**Supplementary Figure 1.** The radiomic profile of recurrent glioblastoma patients (*n*=125) obtained from relative cerebral blood volume (rCBV) map of DSC-MRI.

*Note*: row indicates the patient, and column indicates the radiomic features.

**
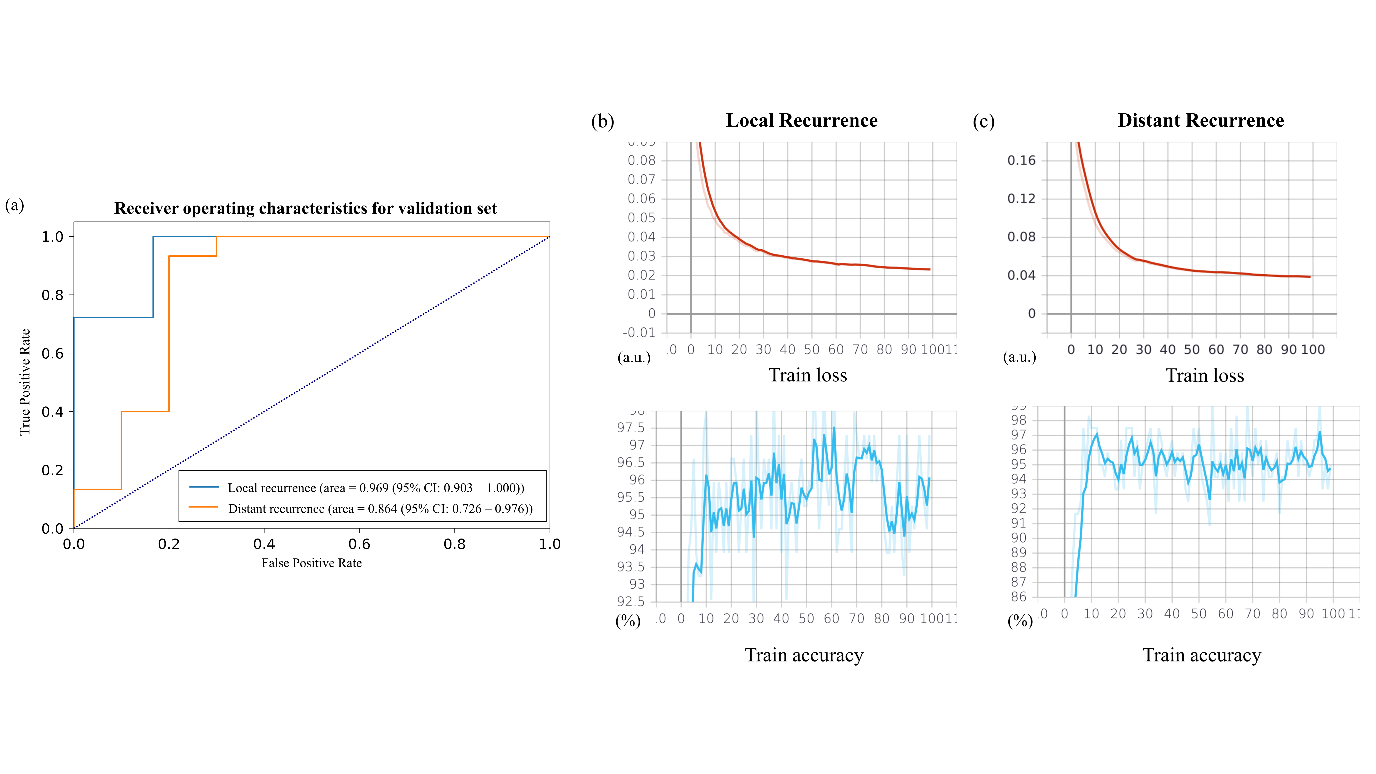
**

**Supplementary Figure 2.** (a) ROC curves, and training curves for loss and accuracy of prediction model for (b) local and (c) distant recurrence. Horizontal axis indicates the epoch of training.

*Note*: units for loss (vertical axis in the upper row of (b) and (c)): arbitrary units (a.u.); and units for accuracy (vertical axis in the bottom row of (b) and (c)): percentage (%).

**
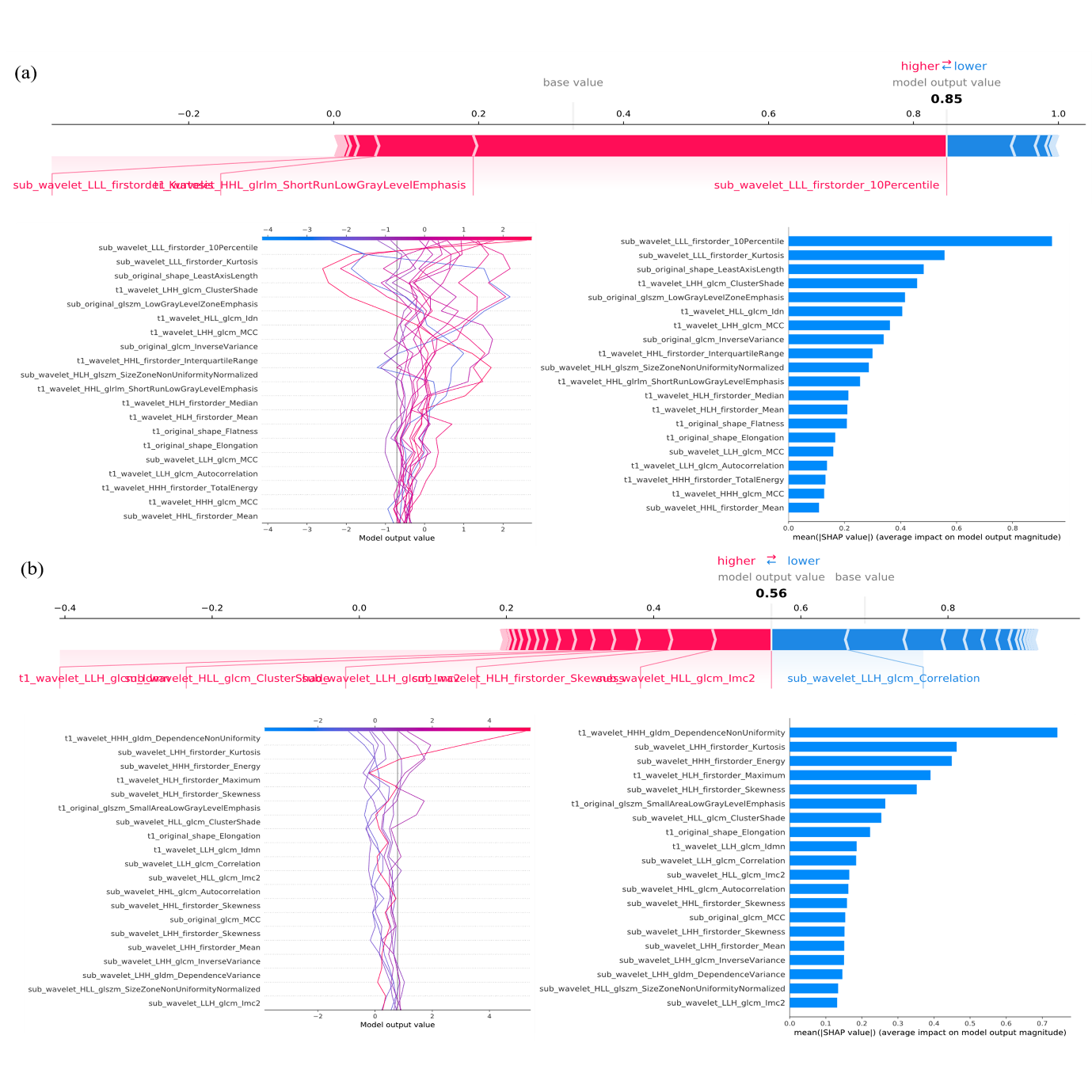
Supplementary Figure 3.** SHAP plots explaining important features for each model to predict (a) local recurrence, and (b) distant recurrence. Each set of SHAP plots consists of three plots: Force plot (top), decision plot (left bottom), and summary plot (right bottom). Note that *sub_wavelet_LLL_firstorder_10Percentile* feature largely increase the model prediction score into the higher direction (arrow) in the force plot of (a), which corresponds to the representative case for local recurrence in Fig. 4a.

**
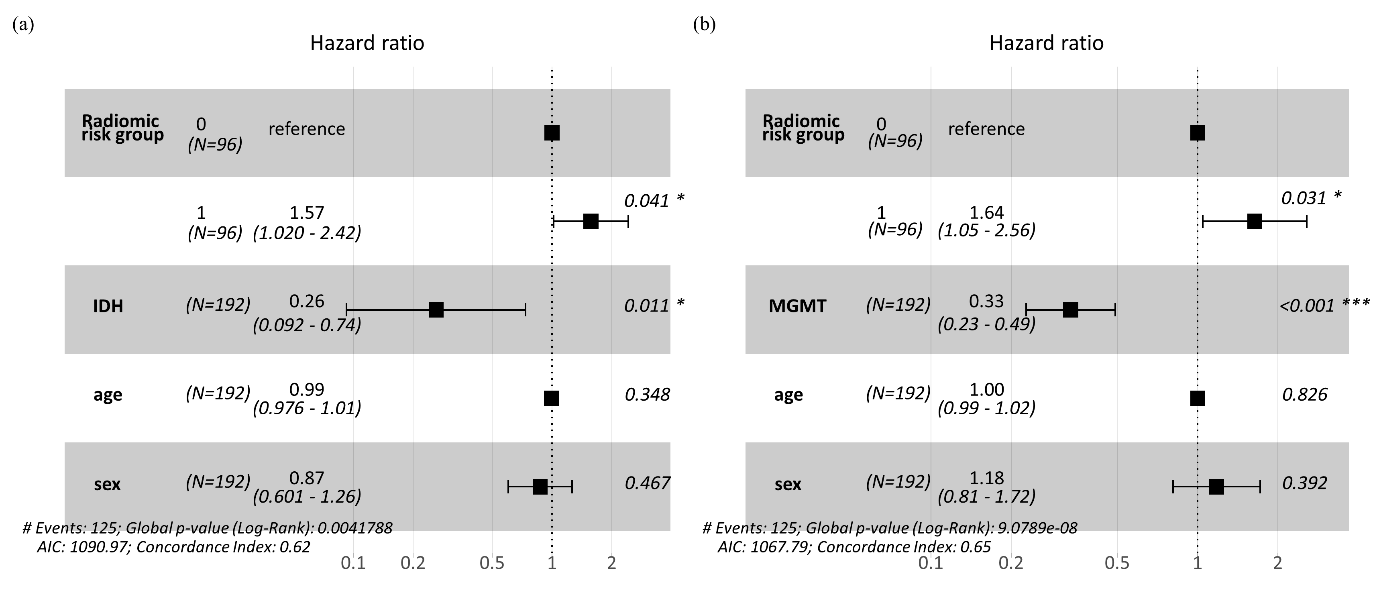
Supplementary Figure 4.** Forest plots for different multivariate Cox-regression models: note that IDH is significant (a) when MGMT status excepted; (b) both radiomic risk group variable and MGMT status are significant when IDH mutation status excepted.

**
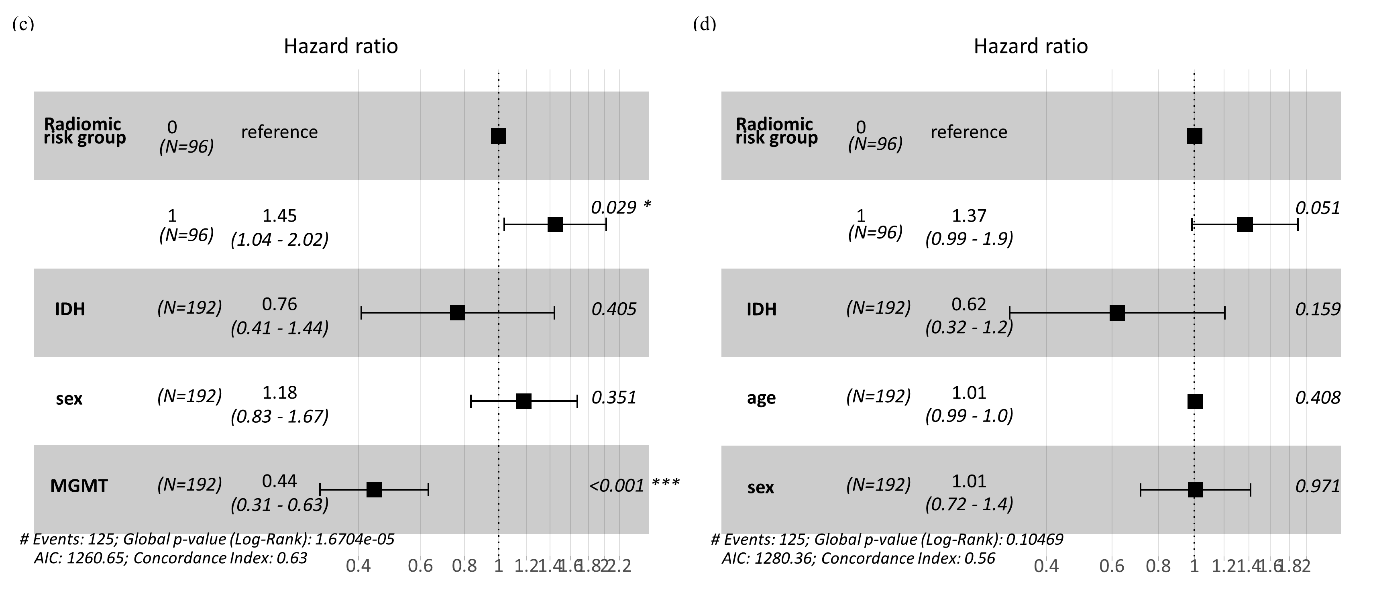
Supplementary Figure 5.** Forest plots for different multivariate Cox-regression models: note that sex variable is not significant both (a) when MGMT status is included, and (b) excepted.


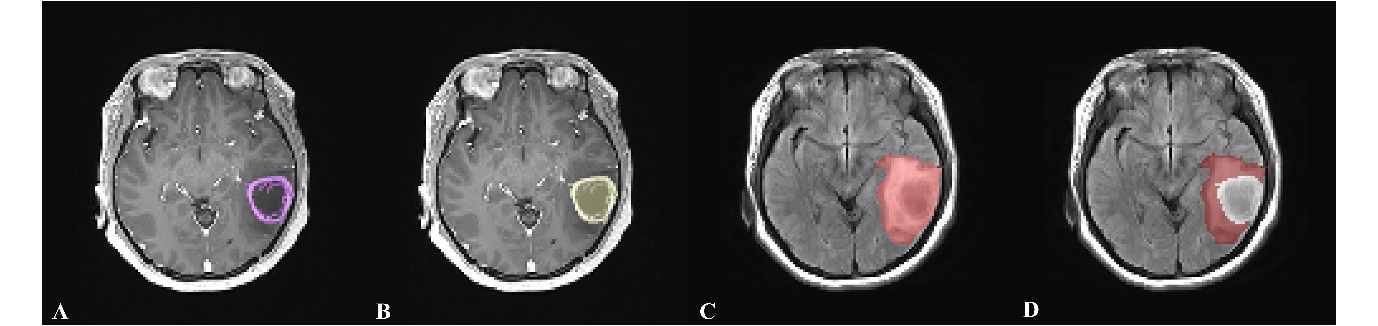


**Supplementary Figure 6.** Segmentation of contrast-enhanced area and non-enhancing T2 high signal intensity area. (A) Contrast enhancing area was drawn semi-automatically on contrast enhanced T1WI, and (B) necrotic portion was added for subtraction. (C) Then, total FLAIR T2 high signal intensity region was drawn. (D) Finally, non-enhancing T2 high signal intensity area was drawn by subtracting enhancing tumor with necrosis portion from total FLAIR high signal intensity region (C-B).


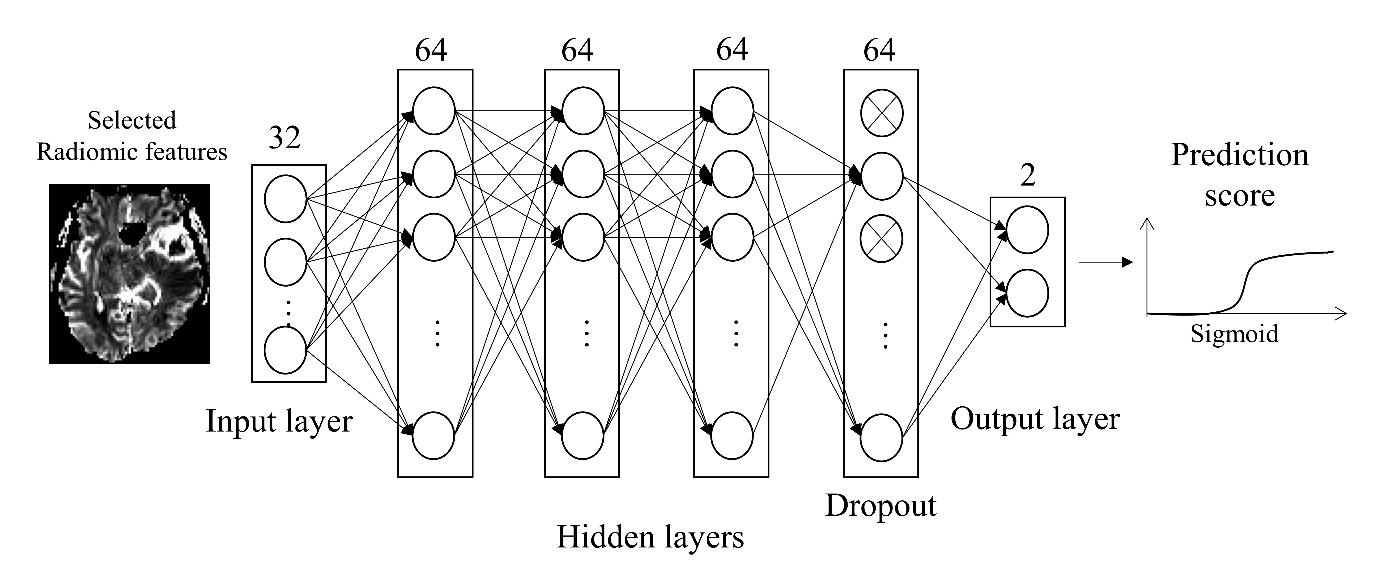


**Supplementary Figure 7.** Model architecture of neural network prediction models: Note that each hidden layer is a simple linear layer, followed by 1-dimensional batch normalization layer, and relu as an activation function. Dropout was applied to final hidden layer to prevent overfitting.

**Supplementary References**

1 Østergaard, L., Weisskoff, R. M., Chesler, D. A., Gyldensted, C. & Rosen, B. R. High resolution measurement of cerebral blood flow using intravascular tracer bolus passages. Part I: Mathematical approach and statistical analysis. *Magnetic resonance in medicine* **36**, 715-725 (1996).

2 Boxerman, J., Schmainda, K. & Weisskoff, R. Relative cerebral blood volume maps corrected for contrast agent extravasation significantly correlate with glioma tumor grade, whereas uncorrected maps do not. *American Journal of Neuroradiology* **27**, 859-867 (2006).

3 Bjornerud, A., Sorensen, A. G., Mouridsen, K. & Emblem, K. E. T1-and T* 2-dominant extravasation correction in DSC-MRI: Part I—theoretical considerations and implications for assessment of tumor hemodynamic properties. *Journal of Cerebral Blood Flow & Metabolism* **31**, 2041-2053 (2011).

4 Wetzel, S. G. *et al.* Relative cerebral blood volume measurements in intracranial mass lesions: interobserver and intraobserver reproducibility study. *Radiology* **224**, 797-803 (2002).

5 Guyon, I., Weston, J., Barnhill, S. & Vapnik, V. Gene selection for cancer classification using support vector machines. *Machine learning* **46**, 389-422 (2002).

6 Chawla, N. V., Bowyer, K. W., Hall, L. O. & Kegelmeyer, W. P. SMOTE: synthetic minority over-sampling technique. *Journal of artificial intelligence research* **16**, 321-357 (2002).

7 Srivastava, N., Hinton, G., Krizhevsky, A., Sutskever, I. & Salakhutdinov, R. Dropout: a simple way to prevent neural networks from overfitting. *The journal of machine learning research* **15**, 1929-1958 (2014).

8 Kingma, D. P. & Ba, J. Adam: A method for stochastic optimization. *arXiv preprint arXiv:1412.6980* (2014).

9 Lundberg, S. M. & Lee, S.-I. in *Advances in neural information processing systems.* 4765-4774.

10 Lundberg, S. M. *et al.* Explainable machine-learning predictions for the prevention of hypoxaemia during surgery. *Nature biomedical engineering* **2**, 749-760 (2018).
